# Heterogeneity in the Developmental Trajectories of Chinese Youth Educational Aspirations: Identifying Predictors and Outcomes

**DOI:** 10.1111/cdev.14234

**Published:** 2025-04-01

**Authors:** Hou Xie, Kaylin Ratner, Suzanne G. Fegley, Michael J. Nakkula

**Affiliations:** ^1^ University of Illinois Urbana‐Champaign Champaign Illinois USA; ^2^ University of Pennsylvania Philadelphia Pennsylvania USA

**Keywords:** Chinese youth, developmental heterogeneity, educational aspirations, growth mixture modeling

## Abstract

This study investigated the development of educational aspirations (EAs) among Chinese youth (*n* = 2228, 48.61% female, 87.66% Han, *M*_age_2010_ = 11.48 years) for 6 years. Five latent classes of EA trajectories were identified. They varied greatly during early adolescence but converged around an associate degree in middle adolescence and beyond and demonstrated high rank‐order stability across the period of study. High academic performance and academic competence (adolescent‐reported) and educational involvement, academic expectations, and family socioeconomic status (parent‐reported) predicted loftier EA trajectories. Consistent EA beyond an associate degree predicted a greater probability of college enrollment in emerging adulthood. Findings are interpreted with respect to China's sociocultural context, a society characterized by high collectivism and regard for academic achievements.

In the second decade of life, young people think about who they are, who they are becoming, and who they can be. This paves the way for the establishment of identity and future possible selves (Erikson 1968; Oyserman and Fryberg 2006). Educational aspirations (EAs) play a key role in this process. Based on prior literature, EAs are defined as adolescents' future‐oriented goals toward a particular educational trajectory or end state, driven by conscious and unconscious motivations, and stemming from their individual and/or collective commitments (Hart 2016). Indeed, constructing an image of a future self reaching a certain level of education can serve as a form of self‐guides (Higgins 1987), setting foundations for realizing other life possibilities, such as pursuing dream careers and moving up the socioeconomic ladder.

As an important facet of possible selves and identity development, youth EA have been studied for decades. Many of these studies have found EA to be a robust predictor of students' later academic achievement (Ansong et al. 2019; Khattab 2015) and higher education enrollment and attainment outcomes (Beal and Crockett 2010). Scholars have also explored factors contributing to EA development, revealing the influential roles of both adolescents' personal characteristics and family contexts (Garg et al. 2002; Rothon et al. 2011; Hartas 2016).

Despite a long history of scholarly attention to youth EA, our current understanding of this topic is derived primarily from cross‐sectional studies in Western societies, along with a limited number of longitudinal studies that focus narrowly on middle to late adolescence (e.g., Liu 2009; Shapka et al. 2012; Lawson et al. 2020). This leaves two significant gaps in the literature. First, given that adolescence is a prolonged period marked by continuous cognitive and psychosocial changes, there is a need for more extensive longitudinal research on EA development that encompasses early adolescence. Second, in East Asian countries like China, education is highly valued and academic achievement is central to cultural notions of success. Yet, EA development among Chinese youth remains largely unexplored territory. Investigation into the development of EA within the Chinese context will increase current knowledge about long‐term patterns of change and stability in youth EA and provide valuable insight into adolescent development in non‐Western societies. Additionally, it could guide practitioners in designing culturally sensitive strategies for supporting youths' educational goals.

To bridge these research gaps, the current study focused on the developmental trajectories of EA among Chinese adolescents. Using data from a nationally representative panel study, our first aim was to probe for possible heterogeneity in the developmental trajectories of EA from early (~11.5 years) to late (~17.5 years) adolescence, or in other words, from elementary school through high school. Beyond this, we also explored how individual and family contextual factors contributed to EA development and investigated how adolescent EA trajectories predicted higher education enrollment outcomes in adulthood.

## 1 Development of EAs During Adolescence

EAs form an important component of one's school‐focused possible selves. Unlike overall self‐concept, which can maintain a stable and enduring internal structure (Markus and Kunda 1986), possible selves are more malleable because they are less dependent on past and present behavioral and societal evidence (Markus and Nurius 1986). Furthermore, applied research has also demonstrated that interventions on adolescents' school‐focused possible selves can effectively facilitate change in desired directions (Oyserman et al. 2002). Although these theoretical accounts and empirical studies have both suggested that adolescents' possible selves might change over time, only a handful of longitudinal studies have examined continuity and change in a possible self dimension like EA. These studies have often focused narrowly on the period after middle adolescence and produced inconsistent findings. For example, some studies have suggested that adolescents' EA generally remains stable, with only a slight increase during high school (Lakshmanan 2004), whereas others have identified a downward trend over time (Lawson et al. 2020). Additionally, Shapka et al. (2012) found that youth had high EA in early high school and after high school but experienced a distinct dip between these bookends. Despite these findings, there has been no systematic discussion about the reasons behind discrepant group‐level developmental patterns.

In the existing longitudinal studies on EA, the primary emphasis was typically on examining the average trajectory of development. This design assumes that all individuals are drawn from a single population with similar developmental pathways. However, when looking for patterns in human development, the classical philosophical law of causality (i.e., similar conditions lead to similar outcomes and different results are caused by dissimilar conditions) does not usually hold (Feiring and Lewis 1987). This suggests the possibility of heterogeneity in EA development. In one case supporting this view, Liu (2009) examined the existence of multiple trajectories of EA development among American teenagers from 10th grade to 2 years after high school. Four distinct trajectory classes were identified from the sample: two classes with increasing trends and two with decreasing trends. Although the study's focus from late adolescence to early adulthood leaves us uncertain of whether these patterns extend from earlier ages, Liu's (2009) investigation highlighted that treating participants as a single group may mask distinctive trajectories of EA development. Therefore, a person‐centered method is crucial to understanding heterogeneity in youth EA development, as well as predictors and outcomes of these pathways.

## 2 Factors Contributing to EA Development

After examining the features of EA trajectories, it is crucial to delve into the key factors contributing to this process to gain a more in‐depth understanding of potential underlying mechanisms. According to Bronfenbrenner's Bioecological Model, the driving force of human development is the reciprocal interactions between a growing person and the environment (Bronfenbrenner and Morris 2006). Oyserman and Markus (1993) similarly asserted that one's pool of possible selves derives from the collaborative interplay between personal inventiveness and sociocultural context. Thus, in terms of EA development, substantial attention has been devoted to the predictive effects of individual and contextual factors.

Demographic characteristics (e.g., gender, urban–rural residence, race, and ethnicity) are the most frequently considered individual factors in prior research. Many studies conducted in Western societies have suggested that girls tend to report higher levels of EA compared to boys of the same age (Rothon et al. 2011; Rampino and Taylor 2013) and exhibit more stable aspiration trajectories over time (Shapka et al. 2012). However, Hannum and Park's (2002) study in rural Gansu, China, observed that boys were more likely to dream of higher education than girls. These contrasting cross‐cultural findings suggest adolescents of the same gender may have different socialization experiences within varying sociocultural contexts. As for the effect of urban–rural residence, earlier studies in the West suggested that rural youth tend to hold lower EA than their non‐rural counterparts (MacBrayne 1987), whereas more recent studies showed upward trends in rural adolescents' EA that were comparable to those of urban adolescents (Witherspoon and Ennett 2011). In the Chinese context, while some research efforts have focused on youth EA in rural areas (for review see Hou 2024), few direct comparisons have been made between rural and urban youth. Concerning the impact of race and ethnicity, studies conducted in Western settings revealed that students from racial/ethnic minority groups typically have higher EA compared to those in racial/ethnic majority groups (Rothon et al. 2011), but these high aspirations might not remain stable (Kao and Tienda 1998). The decline might be attributed to an increased awareness of systemic oppression as youth age, leading them to adjust their aspirations. In China, the population comprises one ethnic majority group (the Han Chinese) and 55 minority groups, yet studies on EA seldom consider the impact of ethnicity. Considered together, it is both reasonable and necessary to investigate (a) whether gender effects uncovered in rural regions in China (typically with more prevalent traditional patriarchal values) can be generalized to broader regions, (b) how urban–rural residence might predict adolescents' EA development, and (c) how EA pathways differ between ethnic minority and Han youth.

In addition to demographic characteristics, at the individual level, prior research has explored the predictive effects of academic performance. Higher academic performance usually corresponds with higher EA (Garg et al. 2002; Widlund et al. 2020), as well as increasing aspirations over time (Liu 2009). Furthermore, students who possess positive personal beliefs in their ability to organize and execute academic tasks typically exhibit a stronger desire to pursue high levels of education (Hartas 2016; Ansong et al. 2019) and have a growing trajectory of EA as they age (Liu 2009). On the one hand, academic performance provides objective feedback, reflecting adolescents' actual learning outcomes and helping them determine their academic standing. Perceived academic competence, on the other hand, is a subjective evaluation of their academic capabilities, reflecting how they view their potential. These two factors complement each other, both serving as potential antecedents of EA development.

Contextually, considerable attention has been dedicated to understanding the impact of family. First, a favorable family socioeconomic status (SES, usually indicated by parents' education level and family income) has nearly always correlated with higher initial EA levels and subsequent growth (Garg et al. 2002; Lawson et al. 2020). Young people from middle and high SES backgrounds benefit from parents' educational backgrounds and financial resources. Parents with higher educational qualifications can leverage their academic experiences to transmit essential competencies and values. Such knowledge is usually not formally taught in schools but is critical for success in the educational system (Bourdieu 1977). This knowledge, combined with tangible financial resources, empowers children's academic pursuits within and beyond school, setting the stage for high EA.

In addition to family SES, other studies have identified positive associations between adolescents' EA and parental educational involvement (Hill and Wang 2015) and expectations (Kirk et al. 2011). Under the Value Perception–Acceptance–Pathway (Cheung and Pomerantz 2015; see also Grusec and Goodnow 1994), parents' involvement in education and high academic expectations convey to their children the high value they place on education. Adolescents may internalize this value and cultivate higher EA.

## 3 EAs and Later Higher Education

Beyond exploring predictors of EA development, another line of research has investigated links between aspirations and educational outcomes. As stated by Markus and Nurius (1986), individuals' representations of possible selves act as a source of motivation: they guide behaviors and attention and lead people to act in identity‐congruent ways. In the domain of education, when specific EAs are cultivated, they function as a self‐guide, prompting young people to put in the necessary effort to achieve the desired degree. Empirical investigations reveal that students with higher EA tend to have higher rates of postsecondary enrollment (Hill and Wang 2015) and attainment (Beal and Crockett 2010). Furthermore, college attendance tends to be more strongly associated with stable and high EA than EA that rises in high school (Ablakwa 2020), underscoring the value of attending to EA early in adolescence.

## 4 EAs in the Chinese Context

To date, most research on adolescent EA has been conducted in Western countries. However, throughout China's extensive history, academic achievement and education have always held a position of great importance. Dating back to the Han Dynasty (206 bce–220 ce), the imperial examination system emphasized the role of academic ability in selecting government officials, leaving an enduring legacy of valuing education within Chinese society. Moreover, under the influence of Confucianism and collectivism, filial piety is regarded as the foremost among traditional virtues. Youth have an obligation to uphold and improve family honor, and such obligation is often manifested in the pursuit of academic excellence and the attainment of advanced degrees.

These cultural values imbue Chinese youth's EAs—as a form of self‐guides—with a more pronounced social significance. Laying the foundation for this idea, Higgins' (1987) Self‐Discrepancy Theory posits two types of self‐guides: the ideal self and the ought self. The ideal self represents the attributes one ideally wishes to possess, whereas the ought self represents the duties that are most often imposed by the social environment. However, theories and empirical evidence from cultural psychology suggest that in collectivist cultural contexts, where achieving interpersonal harmony is a primary goal, obligations and responsibilities may converge with the ideal self, leading the ought self to play a similar, if not more predominant, role in the self‐guide (Higgins 1996; Cheung et al. 2016; Choi 2007). This perspective also aligns with the notion that possible selves “have the potential to reveal the inventive and constructive nature of the self [but also] reflect the extent to which the self is socially determined and constrained” (Markus and Nurius 1986, 954). Therefore, because young people in the Chinese context are often implicitly or explicitly encouraged to dream big for their education, the gradual fusion of ideal‐ and ought‐self aspects of EAs may occur.

Yet, despite the strong emphasis on education and a collective sense of commitment to pursuing education, there is a collision between a large population and limited spots in higher education in contemporary Chinese society. As of 2022 (Ministry of Education of the People's Republic of China 2023), only 60.6% of the eligible youth population had the opportunity to access academically focused high schools. Regarding higher education, 59.6% of eligible youth had the chance to enter college, with 27.9% gaining admission to bachelor's degree programs and the remaining 31.7% pursuing associate degree programs. During adolescence, young people undergo the high school entrance examination (*Zhongkao*) and the national college entrance examination (*Gaokao*), which are upheld as two of the “most critical make‐or‐break exams” in one's life. These two exams directly determine whether a person can secure placements in high schools and colleges, as well as the types of institutions they can attend.

While test scores, typically considered a reflection of students' academic performance, appear to be the most direct and decisive factor in this school recruitment procedure in the Chinese educational system, it is widely acknowledged that less visible factors also influence educational attainment outcomes. EA is among the most significant components (Khattab 2015; Khattab et al. 2022). Although there have been a small number of studies conducted in the Chinese context related to EA, they have primarily concentrated on specific demographics (e.g., rural areas; for review see Hou 2024). This targeted emphasis is valuable, but slows a comprehensive understanding of EA development in the general population of Chinese youth. Among other East Asian countries that emphasize education, such as Japan and Korea, there are even fewer studies on youth EA. The limited research in these areas has focused on the associations of certain psychological and contextual factors, such as a sense of relative deprivation (Matsuyama et al. 2021, in Japan) and maternal employment (Ju and Chung 2000, in South Korea), with EA. This narrow scope of existing studies in Eastern Asian regions, coupled with the uneven distribution of current EA research across various cultural contexts, has created major gaps in a global understanding of youth EA.

## 5 The Present Study

Some liken the educational journey among Chinese youth to a long‐distance race. In this race, some persevere and reach distant finish lines, whereas others withdraw from the competition along the way. In our pursuit to gain a comprehensive understanding of EA development among the general population of Chinese adolescents, we pursued three main research goals. These objectives were largely exploratory in nature.

First, we investigated the existence of distinct EA trajectories from early to late adolescence. Due to the lack of a comprehensive theoretical framework explicitly outlining potential trends in youth EA development, our hypotheses were mainly grounded in empirical evidence from Western contexts. Drawing on Liu's (2009) study of American adolescents, we expected that multiple latent classes of development would be identified, though the exact number of classes could not be anticipated. Further, given that prior research has identified trajectories with varying characteristics (e.g., Lakshmanan 2004; Lawson et al. 2020; Shapka et al. 2012), it was hypothesized that some classes would demonstrate an upward trend in EA over adolescence, others would be characterized by relative stability, and still others would be characterized by decline.

Second, we probed whether individual and family contextual factors in early adolescence predict membership in particular EA classes. We hypothesized that adolescents with greater academic performance, perceived academic competence, parental educational involvement, parental academic expectations, parent education, and family income would be more likely to belong to classes exhibiting higher initial EA levels and/or showing increasing developmental patterns. Regarding the effects of gender, ethnic origin, and urban–rural residence, the investigations remained more exploratory due to the lack of previous studies within the Chinese context. We included these variables as potential predictors due to (1) popular human development theories that emphasize the interaction between individuals and their social contexts; (2) evidence from empirical studies, primarily conducted in the West; and (3) the availability of relevant data in public datasets. Additionally, we probed the interaction of gender and urban–rural residence to expand upon the findings on EA gender differences by Hannum and Park (2002) in rural China.

Lastly, we tested if certain trajectories predicted higher education enrollment in emerging adulthood. We hypothesized that youth in each latent class would have different probabilities of entering higher education, and youth who initialized and/or sustained relatively high levels of EA would have a greater likelihood of enrolling in college.

## 6 Method

### 6.1 Data

Data were derived from the China Family Panel Studies (CFPS), a longitudinal biennial household survey of Chinese communities, families, and individuals designed to support academic and policy research. Launched by the Institute of Social Survey at Peking University in 2010, the first CFPS wave initially sampled 19,986 households (14,960 successful; 33,600 adults and 8990 children). All interviewed households met two requirements: (1) family households were economically independent and (2) at least one member of the family had Chinese nationality. These households were sampled from 25 provinces, municipalities, or autonomous regions, with the population of these regions representing 94.5% of the total population of Mainland China (Xie et al. 2017). Half of these households were generated by oversampling from five large provinces, which formed five independent sub‐sampling frames; another half were drawn from an independent sub‐sampling frame composed of 20 small provinces. Within each of the six sub‐sampling frames, a multi‐stage sampling approach was used: the first stage involved sampling administrative districts/counties, followed by administrative villages/neighborhood communities, and finally households. For stratification, the researchers used administrative units and SES indicated by local Gross Domestic Product (GDP) per capita. This complex approach was chosen to ensure sampling quality and reduce operational costs. Readers seeking further details of the sampling procedure can refer to Xie et al. (2017).

The CFPS 2010 baseline survey was conducted through household visits with trained, face‐to‐face interviewers supported by Computer‐Assisted Personal Interviewing technology. During the household visit phase, the interviewer first presented an information letter about the study to the responsible person of the sampled village/community. Once the support of the village or community was secured, the interviewer visited sampled households and began the interview after obtaining consent from the household respondents. During interviews, individual‐level data (e.g., demographic information, education, and physical and mental conditions) were collected on all members within sampled households, alongside family‐level features (e.g., social and economic activities, daily life). Children under 10 years had their guardians answer all questions on their behalf (i.e., proxy questions), individuals between 10 and 15 years completed a self‐report child questionnaire plus some proxy questions, and individuals aged 16 years and older completed a self‐report adult questionnaire. After the visit, the household received cash compensation, with the amount depending on the number and type of questionnaires completed.

After completing the 2010 baseline survey, all core household members were followed up in biennial surveys, conducted either via computer‐assisted personal or phone interviews. For the current study, data from the first six waves of the CFPS (i.e., 2010, 2012, 2014, 2016, 2018, and 2020) were utilized. Individual and family contextual predictors were measured in 2010 (T1) and developmental trajectories were established based on EA from 2010 to 2016 (T1–T4). Higher education enrollment in emerging adulthood was mainly obtained from the 2020 dataset (T6). For individuals absent from T6, data from 2018 (T5) was used as a supplement.

### 6.2 Participants

The baseline CFPS collected data on 8890 individuals under the age of 15. The analytic sample of this study (*n* = 2228, 48.61% female) included all participants who were between 10 and 13 years (*M*[SD] = 11.48[1.12]) in 2010 (i.e., “early adolescence;” Neinstein et al. 2008). Nine individuals, who failed to provide any EA information from T1–T4, were excluded. Most of the 2010 sample (86.18%) were enrolled in elementary school, 13.11% were enrolled in middle school, and 0.72% were not in school or had missing information. More information on the age and grade of the sample at each wave can be found in Supporting Information (Table S1).

Among the sample, individuals from the Han ethnic group greatly outnumbered those from ethnic minority groups (87.66% vs. 12.12%), and 0.22% did not have information on ethnicity. In addition, 61.49% of the sample resided in rural areas, and 38.50% were in urban areas. Regarding family structure, the average age of mothers (*M*[SD] = 37.84[0.83] years) was slightly younger than that of fathers (*M*[SD] = 39.84[1.19] years). The CFPS did not directly indicate whether a child was from a single‐ or two‐parent family. However, this could be grossly inferred from the marital status of the parents: most of the sample (94.70%) had both parents either married or cohabiting, whereas 2.73% had parents who were both divorced. According to responses to two items asking whether the mother or father lived with the child, most of the youth in the sample (95.57%) resided with at least one parent.

### 6.3 Measurement

The CFPS provides a comprehensive picture of citizens' social, economic, demographic, educational, and health statuses in contemporary Chinese society. Therefore, to capture the desired information efficiently, many variables were measured using scales and items specifically created for the CFPS. These scales were typically concise and straightforward to administer. Additionally, several variables involved in this study were captured by items selected from question groups with a larger number of items. This practice is common in studies utilizing data from large longitudinal projects (cf. Wang et al. 2020). All items appearing on the CFPS are administered in Chinese, but the English version of the questionnaires is provided on the official study website: https://www.isss.pku.edu.cn/cfps/en/index.htm. To facilitate reading, the following presentation of the instruments is grouped by respondents of respective questions.

#### 6.3.1 Questions Answered by Adolescents

##### 6.3.1.1 Educational Aspirations (T1–T4)

Adolescents' EA were measured using one face‐valid item, “What is the minimum level of education you believe that you should attain?” Response options were (1) *No need to go to school*, (2) *Elementary school*, (3) *Middle school*, (4) High school, (5) *2‐ or 3‐year college/Associate degree*, (6) *4‐year college/Bachelor's degree*, (7) *Master's degree*, (8) *Doctoral degree*. Like previous studies (Boxer et al. 2011; Kiang et al. 2015), responses were treated as continuous, with higher values signifying higher EA.

Some may raise concerns about the usage of a single‐item measure to assess EA in this study. However, as highlighted by Allen et al. (2022), the popularity of large panel data in psychological research often necessitates the use of a few or even only one item to capture constructs. This approach helps manage large volumes of data and can improve follow‐up response rates, thereby ensuring high‐quality data. Moreover, empirical evidence has shown that single‐item measures are not inherently indicative of a flawed research design; rather, they can effectively capture the intended construct, especially when the item demonstrates high face validity (Matthews et al. 2022; Allen et al. 2022). In our study, the single item used to measure EAs was designed to be straightforward, unambiguous, and easy to understand. It directly inquired about participants' outlook on the level of education they think they should achieve in the future, aligning well with the construct of EAs as defined in the Chinese cultural context, in which the ideal‐self and ought‐self dimensions often overlap or merge. Moreover, single‐item youth EA assessments have been used in many previous studies on the topic (e.g., Boxer et al. 2011; Kiang et al. 2015).

##### 6.3.1.2 Academic Performance (T1)

Collectively referred to as “cognitive ability,” the CFPS developed two scales to test respondents' word and math skills. However, since these “cognitive ability” scales were constructed by items drawn from the national standard curriculums in Chinese elementary and secondary schools, they are commonly used to index academic performance among individuals in elementary and secondary school (cf., Li and Qiu 2018).

During the household visit interview, all items were asked verbally by the interviewer and answered by the participant. Items were presented in ascending order of difficulty, and respondents were assigned to different scale entry points in accordance with their reported grade level. They were asked to answer each question after their entry point until they failed to answer three consecutive items. Then, their word and math scores were recorded as the rank orders of the last correctly answered item. Since these two sets of items are used in all CPFS surveys, investigators have not disclosed the specific questions to preserve the effectiveness of the test. However, a report on the psychometric properties of these tests is available on the study website (Xu and Luo 2012). For the present study, participants' scores were first standardized within ages. Composite academic performance was then calculated by taking the average of their scores in word and math areas. The Spearman‐Brown coefficient, the most appropriate indicator of the reliability for a two‐item scale (Eisinga et al. 2013), was *r*_SB_ = 0.68 for word and math scores.

##### 6.3.1.3 Perceived Academic Competence (T1)

Perceived academic competence was assessed with two items on a five‐point Likert scale: “How would you rate your academic performance? [(1) not satisfied at all to (5) very satisfied]” and “How excellent of a student do you think you are? [(1) very bad to (5) very good]”. Items were averaged, with higher values indicating greater academic confidence. The Spearman–Brown coefficient of the two items was *r*_SB_ = 0.64.

##### 6.3.1.4 Higher Education Enrollment (T5, T6)

Nearly 10 years after participants initially enrolled in the study, their higher education enrollment status was obtained. Participants were asked if they were currently attending school and, if so, about their current educational level. For those no longer in school, the interviewer inquired about their highest education level attained. Higher education enrollment was coded as (1) for those *enrolled* (including current students and graduates) and (0) for those who *never enrolled*.

#### 6.3.2 Questions Answered by Parents

In the CFPS child questionnaire, questions related to the two variables—parental educational involvement and academic expectations—were designed for the primary caregiver, typically the parents. However, in our sample of 2228 participants, 18.32% of answers (*n* = 399) came from a primary caregiver other than the parents. These responses were excluded from the analysis. Of the remaining available data, 64.13% was reported by mothers and 35.85% was reported by fathers.

##### 6.3.2.1 Parental Education Involvement (T1)

To capture parental education involvement at home, parents were asked to respond to each of three items on a five‐point Likert scale [(1) Always; (2) Usually; (3) Often; (4) Seldomly; and (5) Never]: “How often have you discussed what happens at school with your child since this semester started/last semester?”, “How often did you ask the child to finish homework?”, and “How often did you check the child's homework?”. The items were reverse‐coded before analysis, with higher scores indicating greater parental involvement in children's education. Cronbach's alpha was 0.58, which is considered acceptable given the scale's length (see Xiao et al. 2024).

##### 6.3.2.2 Parental Academic Expectation (T1)

Parental academic expectation was assessed by a single item, “What is the average score, out of a total of 100, that you expect your child to obtain this/next semester (0‐100 points)?” Higher scores indicated greater parental expectations for children's academic achievement.

##### 6.3.2.3 Parents' Education Level (T1)

Parents' education level was assessed by averaging the highest education levels attained by the parents, as collected from parents' individual questionnaires. The scale ranged from (1) Illiterate/Semi‐literate to (8) Doctoral degree.

##### 6.3.2.4 Family Income (T1)

Family income was indicated by the annual net income per capita within the household, using the Chinese Yuan as currency. This information was obtained from a separate household‐level questionnaire that gathered data on the daily life, social interactions, and economic activities of the family.

### 6.4 Data Analytic Strategy

Data cleaning, descriptive statistics, and correlation analyses were performed in R (Version 4.3.1). Growth mixture modeling (GMM) was performed in Mplus 8 (Muthén and Muthén 2017). This procedure allowed us to identify distinctive trajectories of EA development and examine the associations between trajectory class membership, predictors, and the outcome.

GMM was implemented in two stages. First, single‐group latent growth modeling was performed to determine the average baseline model (Ram and Grimm 2009). A no‐growth (intercept‐only) model, a linear growth model, and a quadratic growth model were successively tested to find the best‐fitting representation of the average growth pattern of EA across the entire sample. Model fit was evaluated with the chi‐square test of goodness of fit (*χ* ^2^), comparative fit index (CFI), Tucker–Lewis index (TLI), root mean square error of approximation (RMSEA), and standardized root mean square residual (SRMR). According to Hu and Bentler (1999), CFI and TLI values ≥ 0.95 and SRMR and RMSEA values ≤ 0.08 tend to indicate a good model fit. Additionally, a likelihood ratio test (LRT) was conducted to directly compare the three models. The best‐fitted one was used as the baseline model for subsequent modeling (Ram and Grimm 2009).

Next, based on the baseline model, the three‐step approach of GMM recommended by Asparouhov and Muthén (2014) was used. In Step 1, sub‐trajectories of EA were specified and the optimal number of groups was determined based on information provided by the Bayesian information criterion (BIC), sample‐size‐adjusted BIC (SABIC), Akaike information criterion (AIC), Vuong‐LoMendell‐Rubin LRT (VLMR‐LRT), Lo‐Mendel‐Rubin LRT (LMR‐LRT), Bootstrapped LRT (B‐LRT), and entropy index. For BIC, SABIC, and AIC, lower values tend to suggest a better model fit (Nylund et al. 2007). In contrast, VLMR‐LRT, LMR‐LRT, and B‐LRT are statistical tests to compare adjacent models: a significant *p*‐value indicates a difference between the *k* − 1 and *k*‐class models, with the *k*‐class model typically selected as optimal (Lo et al. 2001). The last index, entropy, checks whether individuals have been accurately classified into the correct latent classes. The value of entropy ranges from 0 to 1, with larger values indicating greater classification accuracy. Entropy above 0.80 is adequate (Lubke and Muthén 2007).

In Step 2, individuals were assigned to the most likely classes derived from Step 1. In Step 3, class membership served as a categorical variable. First, class membership was used as an outcome to reveal the effects of covariates on it through multinomial logistic regression analysis. Second, class membership functioned as a predictor, revealing its association with a distant outcome. Options R3STEP and DCAT were used in Mplus to complete the three steps automatically (Asparouhov and Muthén 2014; Lanza et al. 2013). To facilitate the interpretability of the results, predictors including academic performance, perceived academic competence, home‐based parental education involvement, parental academic expectations, parents' education level, and family income were standardized prior to entry.

### 6.5 Missing Data

The percentage of missing data in EA was low at T1 (2.15%), precluding the examination of differences between missing and non‐missing youth. Attrition increased from T2 to T4, with EA data missing at rates of 24.78% at T2, 34.56% at T3, and 37.97% at T4. Results from Little's MCAR test suggested that missing EA values may not be missing completely at random (MCAR), *χ* ^2^(27) = 58.80, *p* < 0.001; however, the normed chi‐square (*χ* ^2^/df = 2.18) was within the acceptable range (1.0–5.0, Schumacker and Lomax 2004), indicating that they were likely missing at random (MAR) instead. Further, participants with available EA data at each wave from T2 to T4 were compared to those with missing data. There were no significant differences in initial (T1) EA levels between youth with and without missing data at T2 (*t*[2178] = 0.60, *p* = 0.55) and T4 (*t*[2178] = −0.42, *p* = 0.67). However, individuals with missing EA data at T3 had slightly lower initial EA levels compared to those with complete data (*t*[2178] = 2.05, *p* < 0.05). We also used T1 individual and family contextual factors to further identify potential predictors of attrition, applying chi‐square tests for categorical variables and t‐tests for continuous variables. Results are provided in Supporting Information (Table S2). The most notable pattern is that in all three follow‐up waves, participants with missing data had a significantly lower level of academic performance than those with complete data (T2: *t*[2210] = 2.16, *p* < 0.05; T3: *t*[2210] = 3.85, *p* < 0.001; T4: *t*[2210] = 2.09, *p* < 0.05). While observing these missing patterns, it is important to note that such patterns are not necessarily inconsistent with the assumption of MAR, as this assumption allows missingness to be predicted by observed variables in the dataset (Little and Rubin 2019). Consequently, missing values in EA trajectory identification were handled using maximum likelihood with robust standard error (MLR) estimation.

Regarding the T1 predictors of EA trajectory, the percentage of missing data varied across demographic (< 0.22%), adolescent‐reported (0.71% to 1.57%), and parent‐reported (2.73% to 20.20%) information. Regarding T5/T6 enrollment outcomes of EA trajectory, higher education status was obtained from 63.20% of the original sample. With the R3STEP and DCAT options in Mplus, listwise deletion was applied in the analyses concerning EA trajectory and higher education enrollment predictions.

## 7 Results

### 7.1 Heterogenous Developmental Trajectories of EAs

Descriptive statistics and correlations among study variables are provided in Supporting Information (Table S3). Mean scores of EA consistently fell between an associate's and a bachelor's degree at each wave.

To establish a baseline EA trajectory, single‐group growth curve modeling was conducted. A no‐growth (intercept‐only) model (*χ* ^2^[8] = 85.15, CFI = 0.92, TLI = 0.94, RMSEA = 0.07, SRMR = 0.05), a linear growth model (*χ* ^2^[5] = 33.84, CFI = 0.97, TLI = 0.97, RMSEA = 0.05, SRMR = 0.03), and a quadratic growth model (*χ* ^2^[1] = 2.00, CFI = 1.00, TLI = 0.99, RMSEA = 0.02, SRMR = 0.01) were successively tested. The LRT indicated that the linear growth model fit the data significantly better than the no‐growth model (Δ*χ* ^2^ = 51.31, *p* < 0.001) and the quadratic growth model fit the data significantly better than the linear growth model (Δ*χ* ^2^ = 31.84, *p* < 0.001). Therefore, the quadratic growth model was chosen as the baseline model that best represented the changing pattern of EA across the entire sample.

Under the quadratic model, participants demonstrated an initial mean EA (i.e., intercept) of 5.37 (SE = 0.03, *p* < 0.001), a significant negative linear slope of −0.25 (SE = 0.04, *p* < 0.001), and a significant positive quadratic slope of 0.06 (SE = 0.01, *p* < 0.001). As such, this baseline model suggested there was a declining trend for the overall sample in terms of EA from T1 (2010) to T2 (2012), followed by a slight upturn from T2 (2012) to T4 (2016). There was a significant negative covariance between the linear and quadratic slopes (*ψ*_SS_ = −0.13, SE = 0.06, *p* < 0.05): individuals who initially experienced a higher rate of change in EA tended to show weaker changes in that trajectory over time. However, the covariances between the intercept and both the linear slope (*ψ*_IS_ = −0.22, SE = 0.18, *p* = 0.23) and the quadratic slope (*ψ*_IS_ = 0.03, SE = 0.05, *p* = 0.46) were not significant. In addition, there was also significant estimated variation in the intercept (*σ* ^2^ = 0.93, SE = 0.17, *p* < 0.001), linear slope (*σ* ^2^ = 0.47, SE = 0.22, *p* = 0.04), and quadratic slope parameters (*σ* ^2^ = 0.04, SE = 0.02, *p* = 0.02), suggesting individual differences in initial mean levels and rates of change across the period of study. Thus, it was legitimate to search for unobserved subgroups in these growth trajectories.

Using the baseline quadratic model, a series of GMMs ranging from one to seven sub‐trajectory groups were modeled to determine the optimal number of latent classes. As suggested by Johnson (2021), both class‐invariant models (variance and covariance of intercepts and slopes constrained to equality within each class) and class‐variant models (variance and covariance estimated freely in each class) were tested. However, in the class‐variant sequence, the models failed to converge starting from the 3‐trajectory solution. Therefore, we focused our efforts on identifying class‐invariant solutions.

Judging from several fit indices (Table 1), the 5‐trajectory class‐invariant solution was selected as the optimal solution. The AIC, BIC, and SABIC values decreased as group numbers increased, but the VLMR‐LRT and LMR‐LRT returned nonsignificant values starting at the 6‐trajectory solution. The entropy index value of the 5‐trajectory model was 0.98, suggesting that most individuals could be accurately assigned to the correct latent classes.

**TABLE 1:** Fit indices of 1‐trajectory to 7‐trajectory models.

Group	Log‐likelihood	AIC	BIC	SABIC	VLMR‐LRT	LMR‐LRT	BLRT	Entropy
1	−11,169.28	22,358.56	22,415.65	22,383.87	N/A	N/A	N/A	N/A
2	−10,931.63	21,891.26	21,971.19	21,926.71	< 0.001	< 0.001	< 0.001	0.78
3	−10,667.61	21,371.22	21,473.98	21,416.79	< 0.001	< 0.001	< 0.001	0.92
4	−10,465.24	20,974.48	21,100.08	21,030.18	< 0.001	< 0.001	< 0.001	0.91
**5**	**−10,179.01**	**20,410.01**	**20,558.44**	**20,475.84**	**< 0.005**	**< 0.005**	**< 0.001**	**0.98**
6	−9206.22	18,472.43	18,643.70	18,548.38	0.32	0.33	< 0.001	0.98
7	−6627.26	13,322.52	13,516.62	13,408.59	0.88	0.88	< 0.001	0.98

*Note:* Bold indicates the selected model. Due to the presence of the Heywood case in the selected 5‐class models (see the table notes in Table 2 for more information), the intercept variances of all models from 2 to 7 classes are fixed to zero as approximate likelihood ratio tests are only applicable for comparing models with the same variance–covariance specification (Johnson 2021).

**FIGURE 1:**
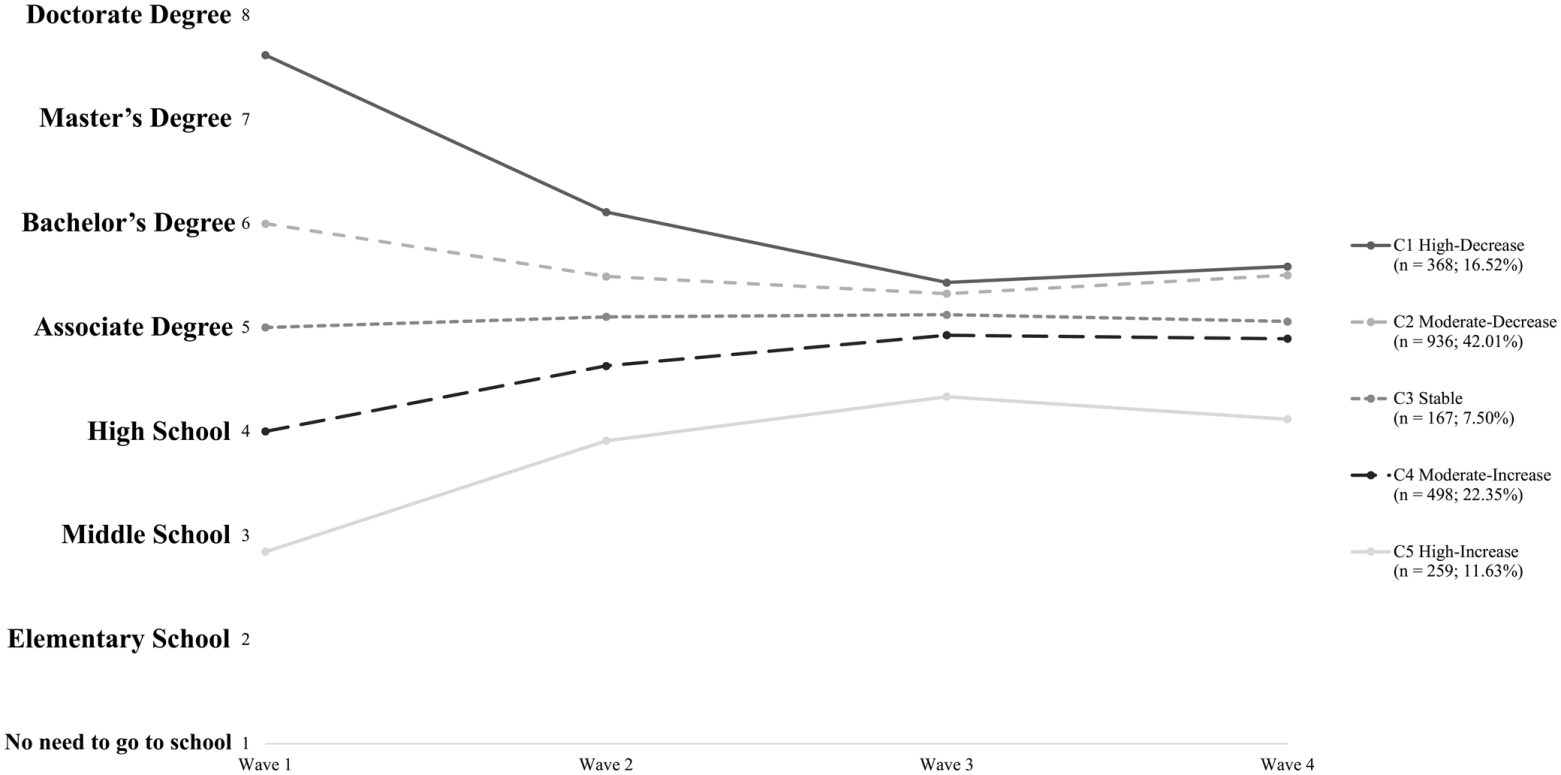
Trajectories of the five latent classes.

The model‐implied EA trajectories for each of the five classes are visualized in Figure 1. In Class 1 (High‐decrease class; *n* = 368; 16.52% of the sample), youth were characterized by high EA in early adolescence (i.e., earning a graduate‐level degree) with a sharp decline in their aspirations from T1 to T3 followed by a slight increase until T4. In Class 2 (Moderate‐decrease class; *n* = 936, 42.01% of the sample), individuals began with a medium‐high level of EA that modestly declined before increasing. Class 3 (Stable class; *n* = 167; 7.50% of the sample) consisted of those who reported associate‐degree‐level EA at T1 with minimal fluctuation across the four target waves. In Class 4 (Moderate‐increase class; *n* = 498; 22.35% of the sample), individuals' initial EA were medium‐low. However, these youth tended to increase in terms of their EA from T1 to T3 before remaining stable over the next 2 years. At last, Class 5 (High‐increase class; *n* = 259; 11.63% of the sample) consistently had the lowest EA compared to the other four groups. This trajectory slowly increased from T1 to T3, but declined in the final 2 years. Table 2 shows intercept and slope parameter estimates for each class, as well as variances and covariances. Except for the smallest group (Stable; Class 3), all classes had significant linear and quadratic slope estimates. This suggests that most of the sample experienced varying degrees of change in EA over adolescence.

**TABLE 2:** Parameter estimates for the selected five‐class growth mixture model.

Parameter	Class 1	Class 2	Class 3	Class 4	Class 5
Mean					
Intercept	7.62***	6.00***	5.00***	4.01***	2.84***
Linear slope	−1.93***	−0.68***	0.15	0.80***	1.39***
Quadratic slope	0.42***	0.17***	−0.04	−0.17***	−0.32***
Variances					
Intercept	0.00	0.00	0.00	0.00	0.00
Linear slope	0.70***	0.70***	0.70***	0.70***	0.70***
Quadratic slope	0.04*	0.04*	0.04*	0.04*	0.04*
Covariances					
Linear slope, quadratic slope	−0.17***	−0.17***	−0.17***	−0.17***	−0.17***

*Note:* A Heywood case emerged in the initial model specification, where the variance of the intercepts was estimated as −0.04. Therefore, the variance of the intercept was consequently fixed to zero (see Dillon et al. 1987), making the subsequent covariances with the intercept also zero.

* *p* ≤ 0.05.

*** *p* ≤ 0.001.

Taken together, adolescents assigned to the five latent classes had distinct developmental trajectories of EA. Their trajectories, stemming from widely scattered starting points, gradually converged to around an associate degree between T1 and T3. Inter‐trajectory differences were minimized at T3 (mean age ~15.5) before becoming more variant again by T4 (mean age ~17.5).

### 7.2 Individual and Family Contextual Factors on Latent Class Membership

Individual and family contextual factors were added to the selected 5‐trajectory model as auxiliary variables to predict latent class membership. Odds ratios (ORs) and multinomial logistic regression coefficients for each pairwise class comparison appear in Table 3. In terms of demographics, boys were more likely to be classified into the High‐decrease class (Class 1) than the Moderate‐decrease class (Class 2). Ethnic minority youth were over twice as likely as Han youth to be classified into the Stable class (Class 3) compared to the Moderate‐increase class (Class 4). Regarding residence, no significant differences were observed between urban and rural youth. The interaction between gender and urban–rural residence was further explored through a multinomial logistic regression model that only considered these two variables. This is because the interaction effect in logistic regression is non‐linear, which will be conditional on the levels of other variables if they are included in the model (Ai and Norton 2003). By including only gender and urban–rural residence, the model could isolate the interaction, making the effect easier to interpret. However, the analysis revealed no significant interaction effects, indicating that the predictive effect of gender on group membership is relatively consistent across both urban and rural settings. Details on the interaction analysis can be found in Table S3.

**TABLE 3:** The associations between individual and family contextual factors and class membership.

Variable	Class 1 vs. Class 2			Class 1 vs. Class 3			Class 1 vs. Class 4			Class 1 vs. Class 5			Class 2 vs. Class 3		
	High‐decrease vs. moderate‐decrease			High‐decrease vs. stable			High‐decrease vs. moderate‐increase			High‐decrease vs. low‐increase			Moderate‐decrease vs. stable		
	Estimates	SE	OR	Estimates	SE	OR	Estimates	SE	OR	Estimates	SE	OR	Estimates	SE	OR
Gender (F = 0, M = 1)	−0.35*	0.15	0.71	−0.25	0.22	0.78	−0.18	0.17	0.83	−0.36	0.22	0.70	0.10	0.20	1.10
Ethnic (Han = 0, minority = 1)	−0.12	0.26	0.89	0.41	0.33	1.51	−0.36	0.29	0.70	−0.23	0.33	0.79	0.53	0.28	1.69
Residence (rural = 0, urban = 1)	0.13	0.17	1.14	−0.21	0.26	0.81	−0.12	0.20	0.88	0.05	0.26	1.05	−0.34	0.23	0.71
Academic performance	−0.26*	0.11	0.77	−0.09	0.16	0.91	−0.26*	0.12	0.77	−0.72***	0.14	0.49	0.16	0.15	1.18
Perceived academic competence	−0.31***	0.08	0.74	−0.36**	0.11	0.70	−0.48***	0.09	0.62	−0.72***	0.11	0.49	−0.05	0.09	0.95
Parental involvement	−0.24*	0.09	0.78	−0.30*	0.12	0.74	−0.27*	0.10	0.76	−0.45***	0.12	0.64	−0.06	0.11	0.94
Parental academic expectation	−0.17	0.10	0.84	−0.26	0.14	0.77	−0.38**	0.11	0.68	−0.46***	0.13	0.63	−0.08	0.11	0.92
Parents' educational level	−0.17*	0.09	0.84	−0.34*	0.13	0.71	−0.43***	0.10	0.65	−0.82***	0.16	0.44	−0.17	0.11	0.84
Family income	0.05	0.07	1.05	0.10	0.09	1.11	0.06	0.10	1.06	−0.48*	0.24	0.62	0.05	0.07	1.05

Variable	Class 2 vs. Class 4			Class 2 vs. Class 5			Class 3 vs. Class 4			Class 3 vs. Class 5			Class 4 vs. Class 5		
	Moderate‐decrease vs. moderate‐increase			Moderate‐decrease vs. high‐increase			Stable vs. moderate‐increase			Stable vs. high‐increase			Moderate‐increase vs. high‐increase		
	Estimates	SE	OR	Estimates	SE	OR	Estimates	SE	OR	Estimates	SE	OR	Estimates	SE	OR
Gender (F = 0, M = 1)	0.16	0.14	1.18	−0.02	0.19	1.10	0.07	0.21	1.07	−0.11	0.24	0.90	−0.18	0.20	0.84
Ethnic (Han = 0, minority = 1)	−0.25	0.22	0.78	−0.12	0.27	1.69	−0.77*	0.30	0.46	−0.64	0.33	0.53	0.13	0.28	1.14
Residence (rural = 0, urban = 1)	−0.26	0.16	0.78	−0.08	0.23	0.71	0.09	0.25	1.09	0.26	0.30	1.30	0.17	0.25	1.19
Academic performance	0.00	0.09	1.00	−0.46***	0.11	1.18	−0.17	0.15	0.85	−0.62***	0.17	0.54	−0.46***	0.12	0.63
Perceived academic competence	−0.17*	0.07	0.84	−0.42***	0.09	0.95	−0.12	0.10	0.89	−0.36**	0.11	0.70	−0.24*	0.09	0.78
Parental involvement	−0.03	0.07	0.97	−0.20*	0.10	0.94	0.03	0.11	1.03	−0.15	0.13	0.87	−0.17	0.10	0.84
Parental academic expectation	−0.21**	0.07	0.81	−0.29**	0.09	0.92	−0.12	0.11	0.89	−0.20	0.12	0.82	−0.08	0.09	0.92
Parents' educational level	−0.26**	0.08	0.77	−0.65***	0.15	0.84	−0.09	0.12	0.92	−0.48**	0.17	0.62	−0.40*	0.15	0.67
Family income	0.01	0.09	1.01	−0.53*	0.24	1.05	−0.04	0.11	0.96	−0.58*	0.24	0.56	−0.54*	0.24	0.58

*Note:* Reference classes are listed on the left side.

* *p* ≤ 0.05.

** *p* ≤ 0.01.

*** *p* ≤ 0.001.

In terms of other predictors, adolescents with higher academic performance, perceived academic competence, parental educational involvement, parental academic expectations, parent education, and family income were generally more likely to belong to the High‐decrease (Class 1) and Moderate‐decrease (Class 2) classes compared to the High‐increase class (Class 5). Put simply, individuals with higher scores in these areas were more inclined to be part of classes where they started with medium to high levels of EA, which persisted above an associate degree through adolescence despite some decline. Conversely, higher scores on these variables reduced the likelihood of belonging to the High‐increase class (Class 5), which comprised adolescents initiating the study with the lowest EA that increased only to middle and high school degree levels over the 6 years of observation.

It is worth noting that among the two indicators of family SES, parents' education and family income had divergent associations with class membership. Significant effects of parents' education were found in eight out of 10 pairwise comparisons: adolescents with more educated parents were more likely to belong to trajectories characterized by medium to high EA. In contrast, family income was a much weaker predictor of trajectory. Adolescents from wealthier families were less likely to be in the High‐increase class (Class 5) than in the other four.

### 7.3 Predicting Higher Education Enrollment Outcome From EA Trajectory

Class membership, or EA trajectory, was significantly associated with higher education enrollment status after age 18 (*χ* ^2^[4] = 155.67, *p* < 0.001). In each of the five classes, the probabilities of individuals eventually entering higher education were 0.68, 0.52, 0.60, 0.49, and 0.18, respectively. As seen in Table 4, adolescents who expressed aspirations beyond an associate degree throughout early to late adolescence (i.e., Classes 1–2) were significantly more likely to enter higher education than those who did not (i.e., Classes 4–5).

**TABLE 4:** The associations between EA trajectory and higher education enrollment outcome.

	*χ* ^2^ (df)	*p*
Overall test	155.67 (4)	< 0.001
Class 1 vs. Class 2	2.64 (1)	0.10
Class 1 vs. Class 3	2.41 (1)	0.12
Class 1 vs. Class 4	20.66 (1)	< 0.001
Class 1 vs. Class 5	120.60 (1)	< 0.001
Class 2 vs. Class 3	0.29 (1)	0.59
Class 2 vs. Class 4	14.02 (1)	< 0.001
Class 2 vs. Class 5	126.30 (1)	< 0.001
Class 3 vs. Class 4	3.41 (1)	0.07
Class 3 vs. Class 5	50.43 (1)	< 0.001
Class 4 vs. Class 5	49.76 (1)	< 0.001

### 7.4 Sensitivity Analyses

Given the complexity of the modeling procedures employed in the current study, we performed a series of sensitivity analyses to validate the robustness of our findings. This included testing alternative model specifications, utilizing different missing data handling techniques, replicating the trajectory identification procedure on a subsample, and conducting separate analyses for female and male youth. With only slight variations in the recovered trajectories partitioned by gender, we found similar patterns of results across other modeling adjustments. Full descriptions, tables, and figures regarding these sensitivity analyses are available in the Supporting Information.

## 8 Discussion

In the current study, data from a nationally representative and longitudinal household survey were used to investigate trajectories of EA development among Chinese youth from early to late adolescence. This is relevant, as adolescence is a critical period for young people to navigate life possibilities and reconcile possible selves, yet our current knowledge about EA development is constrained to only portions of adolescence in largely Western contexts. We identified five latent classes of developmental trajectories characterized by distinct initial EA statuses and rates of change across adolescence. The results also revealed that individual and family contextual factors, such as gender, ethnicity, academic performance, perceived academic competence, parental educational involvement, parental academic expectations, parent education, and family income, had differential predictive effects on trajectory membership. Furthermore, individuals in different trajectory groups had varying probabilities of enrolling in higher education. These findings partially align with previous studies conducted in Western societies but also highlight some unique developmental characteristics specific to Chinese adolescents.

### 8.1 Heterogeneity in EA Developmental Trajectories

Five distinct trajectories (High‐decrease [Class 1], Moderate‐decrease [Class 2], Stable [Class 3], Moderate‐increase [Class 4], and High‐increase [Class 5]) emerged, suggesting previously unobserved heterogeneity in EA development among Chinese adolescents. Most adolescents experienced varying degrees of change in their EA, but the differences between subgroups diminished over time. Importantly, however, there was no crossover between any of the five trajectories, implying relative stability in rank‐order despite fluctuations in absolute EA value over time. In other words, adolescents who initially harbored high EA tended to maintain high ambitions compared to their peers in the following years. Conversely, those who began the study with low EA consistently lagged behind their counterparts. These results expand the developmental pattern identified in a sample of U.S. high school students (Liu 2009), which showed that late adolescents' EA either increased or decreased over time, but their relative positions remained largely the same. The current study goes further, demonstrating that such stability extends and persists from early adolescence.

When looking at the characteristics of EA trajectories, we noted a considerable initial dispersion: adolescents in the first class dreamed of graduate‐level degrees, whereas those in the fifth class barely aspired to middle school completion. However, as these young people grew up, their EA gradually converged toward middle ground. By 2014 (T3), with an average age of 15.5 years, all five classes fell between finishing high school education and getting a bachelor's degree. While there is no direct theoretical explanation for these patterns, we can draw a few insights from the field of career development. Between preteen years and young adulthood, individuals' aspirations for future vocational choices typically pass through three stages: fantasy, tentative, and realistic (Ginsberg et al. 1951). The current findings indicate a similar developmental process in EA. In 2010 (T1), most of the sample was in elementary school and only held vague perceptions of the purpose and essence of education. Hence, their visions of the future largely stemmed from fantasy. Some of them had not yet realized the difficulties and challenges associated with pursuing higher education, leading to overly ambitious attitudes. At the same time, some held very low EA as they had not recognized the value of education, envisioning a world where people could secure good job opportunities and lead comfortable lives without a decent education. However, within the Chinese school system, students would experience a sharp increase in learning difficulty and academic pressure during the transition from elementary to middle school. This transition may have brought them closer to the real world, fostering a clearer self‐perception of their academic competence and awareness of the necessity of education. Therefore, from 2010 (T1) to 2014 (T3), most individuals in the sample gradually adjusted their EA to align with reality.

Interestingly, from 2014 (T3) to 2016 (T4), a divergence re‐emerged among the five trajectories, and the disparities between classes expanded. One possible explanation for this trend is that during these 2 years, most participants completed middle school but only a subset had the opportunity to enroll in academically focused high school. Academically focused high schools are a stepping stone toward higher education—teens fortunate enough to gain admission to academically focused high schools may have become more optimistic about their chances of going to college and naturally elevated their EA once again. Conversely, the opposite would occur for adolescents who aspired modestly and discontinued their education after middle school or enrolled in a vocational high school.

Regarding latent class sizes, we noticed an uneven distribution of adolescents. In the first three classes, which accounted for over 65% of the entire sample, adolescents' EA never dropped below an associate degree. Given the Chinese culture's strong emphasis on education, it is unsurprising that more than half of young people viewed higher education as an unquestionable pathway to a secure and prosperous future. In the second latent class alone, over 40% of the sample was represented. In this class, adolescents initially aspired to achieve a bachelor's degree but later shifted between pursuing a bachelor's and an associate degree. This may reflect a normative trend within contemporary China, a developing country where the prevalence of graduate‐level education remains relatively low due to economic and educational resource limitations. Therefore, fewer individuals with advanced degrees could serve as role models for youth. Furthermore, during middle and high school years, many teachers and parents sacralize attending college as a form of salvation, causing adolescent students to aspire to undergraduate studies and defer ambitions for advanced degrees. It is plausible that aspirations for graduate‐level education emerge mainly *after* the attainment of undergraduate goals. Future research could help confirm this hypothesis.

### 8.2 Predictors and Outcomes of EA Trajectories

First, compared with girls, boys were more likely to belong to the group beginning with the highest EA but followed by a dramatic decline. Girls' initial EA tended to be more conservative, and subsequent fluctuations tended to be milder. This pattern is partially consistent with the findings of Shapka et al. (2012), who reported that Canadian boys underwent more pronounced EA changes during adolescence compared to girls. This might be due to the societal narrative favoring assertiveness and courage in boys, which empowers them to explore a wider range of educational possibilities and results in fluctuating aspirations as they weigh different options. In contrast, gender norms often guide girls toward stable, conventional careers with lower educational requirements from an early age. This steering may lead to a perception of limited options, contributing to greater stability and conservatism in girls' EA development.

Second, and consistent with recent Western research (e.g., Witherspoon and Ennett 2011), we failed to find evidence of urban–rural differences in EA class membership. This may support Haller and Virkler's (1993) argument that the urban–rural EA discrepancy found in prior studies largely stems from divergence in family SES (we explore this possibility in more depth below). We also failed to find evidence of an interaction between gender and urban–rural residence status, suggesting similar gender effects across urban and rural areas. This could be due to our study's broad geographic scope, covering regions with varying urban–rural disparities. In economically prosperous regions, the economic reforms since the 1980s have markedly narrowed the gaps in value systems between urban and rural residents. Meanwhile, in inland regions such as Gansu, where Hannum and Park (2002) conducted their research, significant urban–rural disparities persist. For example, rural populations in these areas often adhere to more traditional patriarchal cultural values. The wide geographic coverage of our study may have caused regional variations to offset one another, masking the urban–rural difference in gender in certain areas and resulting in a nonsignificant interaction.

Third, this study found modest evidence for ethnic differences in EA development, with only one significant difference observed. Specifically, ethnic minority adolescents were approximately twice as likely to be classified into the Stable class (Class 3) compared to the Moderate‐increase class (Class 4). This finding indicates that, compared with peers from the Han group, ethnic minority adolescents were more likely to display a stable EA developmental pattern with an intermediate level, which differs from results observed in Western societies where ethnic minority students face a greater risk of lowering their aspirations as they age (Kao and Tienda 1998). This difference may be explained by the fact that most Chinese ethnic minority groups do not have readily discernible physical characteristics that distinguish them from the Han majority, resulting in fewer conspicuous social injustices than those experienced by Western ethnic‐racial minority groups. Ethnic minority youth in China may not encounter the same obstacles that impede their academic success, so they can maintain their academic goals. However, barriers do persist as many ethnic minority communities are concentrated in underdeveloped regions of China, where the quality of education and people's educational attainment often fall below the national average. This social context may constrain these youth's ability to envision the full scope of future educational possibilities, leading to a tendency toward conservative aspirations. In other words, despite holding aspirations for higher education, they perceive an associate degree as both satisfactory and realistic.

Fourth, the results concerning academic performance and perceived academic competence partially aligned with our expectations. In general, higher academic performance and greater perceived competence during early adolescence were linked to trajectories with high initial EA. However, since these high initial levels were typically followed by a downward trend, greater academic performance and perceived competence are not necessarily precursors to a rise in EA. Our findings also indicate that perceived academic competence appeared to be a stronger predictor of EA development trajectories than actual academic performance, as evidenced by perceived academic competence having discriminative effects cross more pairwise class comparisons. This implies that EA may be more closely tied to subjective factors, such as adolescents' personal beliefs and attitudes toward their academic ability, which aligns with Markus and Nurius's (1986) assertion that possible selves represent a part of the self‐concept that is generally more flexible and less dependent on objective realities. These findings appear to be good news for practitioners as well, signifying that students with less‐than‐ideal academic records can still aspire to access higher education if provided with support to believe in their capacity for improvement and success.

Fifth, regarding family contextual influences, adolescents who experienced greater parental educational involvement and parental academic expectations were more likely to report higher levels of EA over time. Although similar results have been found in Western samples (e.g., Hill and Wang 2015), these parental influences should be interpreted from a perspective of Chinese cultural values. In a collectivist society that stresses interdependence, higher education not only serves as a vehicle for students' personal success but also symbolizes honor for parents and the extended family. Therefore, the pursuit of an advanced degree is endowed with greater meaning; it is no longer merely an individual choice but a shared family desire. According to the Value Perception‐Acceptance‐Pathway (Cheung and Pomerantz 2015; see also Grusec and Goodnow 1994), when parents actively participate in their children's learning activities and hold high academic expectations, they are implicitly transmitting their emphasis on education and instilling these values in children. This process also nurtures and reinforces the sense of responsibility and obligation in children, inspiring them to reciprocate their parents' support by dedicating themselves to academic work and striving for college. This dynamic also reflects the Chinese perspective on EAs, where one's hope for educational attainment is seamlessly intertwined with a deep sense of responsibility and duty.

Next, the results revealed that two indicators of family SES have different effects on EA development. Consistent with hypotheses, children with more educated parents were more likely to be classified into trajectories characterized by high EA. During adolescence, parents are crucial role models for their children, shaping their perceptions of adult life. For example, when parents have higher education, their children are more aware of educational possibilities and exposed to the benefits of education (e.g., social privilege and income potential). In contrast, the effect of family income appears to be more specific, primarily distinguishing the High‐increase class (Class 5) from the other four classes. That is, individuals with the lowest level of EA over time tended to come from families with lower incomes. Adolescents from less affluent households may feel a greater urgency to contribute to their families at an early age. In such cases, teenagers may hope to leave school early to search for employment, prioritizing immediate financial gains over further education.

Finally, our examination of higher education enrollment indicated that adolescents belonging to the first two classes (i.e., High‐decrease and Moderate‐decrease) were significantly more likely to enter higher education than those in the last two classes (i.e., Moderate‐increase and High‐increase). These findings align with Markus and Nurius's (1986) theory of possible selves, which implies that EA could serve as a motivational force, encouraging behaviors that narrow the distance between one's current self and future selves. Meanwhile, the consistent rank‐order of EA throughout adolescence provides a key insight: it is the early aspirations, rather than the subsequent changing trends, that predict long‐term higher education enrollment. Although individuals in the latter two classes (i.e., the Moderate‐increase and the High‐increase) raised their EA at a later stage, this upward shift could not entirely compensate for lower initial levels, consistent with prior research in shorter intervals (Ablakwa 2020). As a result, they were still significantly less likely to enter college compared to the individuals in the first two groups.

### 8.3 Limitations and Future Directions

Despite providing a comprehensive picture of Chinese adolescent EA development, several limitations should be considered when interpreting these results. First, when examining the predictive roles of individual and family contextual factors on EA trajectory class membership, we only utilized data from the first wave of measurement for the predictors. However, individuals' academic performance, perceived academic competence, parental educational involvement, and parental academic expectations may not remain steady during adolescence. Changes in these aspects could also impact the development of EA and, conversely, adolescents' EA might influence the changes in these factors. Future studies could employ cross‐lagged panel designs to explore bidirectional effects between the development of EA and other personal and family factors.

Another limitation related to these predictor variables is that many of them were not measured using well‐established instruments, resulting in some cases of acceptable but less‐than‐ideal reliability. Replication of this study with well‐established measures might be needed to further validate the results.

Additionally, in terms of ethnic group effects, we acknowledge that our sample composition (87.66% Han) potentially created some challenges for robustly predicting EA trajectories. While this distribution largely reflects China's national demographic composition, uneven sample sizes might have weakened the statistical power and precision of our ethnic comparisons. Future research would benefit from targeted sampling strategies to recruit larger samples of ethnic minority students, enabling a robust assessment of whether the observed differences in EA between Han and ethnic minority youth reflect true group differences.

Finally, as suggested by Robinson et al. (2018), GMM is a data‐driven method. Using this kind of method, the decision regarding model selection is primarily guided by empirical evidence present in the data rather than by the existing theoretical frameworks or assumptions. Furthermore, we recognize that the observed funnel shape of the five trajectories may be attributable to statistical artifacts, like regression toward the mean. However, current analytical methods do not allow us to detect or fully rule out this possibility. Replication with additional samples is therefore necessary to verify the generalizability and veracity of our findings. Nevertheless, we still want to emphasize the rank‐order stability across the EA trajectories found in this study. This stability indicates that, even if the convergence of trajectories may be due to statistical artifacts, individual differences remain remarkably consistent. This consistency may underscore the lasting impact of early‐onset individual and family contextual factors examined in this study, which help explain why some individuals maintained higher or lower EA compared to their peers over time.

## 9 Conclusions and Implications

Using a large longitudinal sample, this study marks the first investigation into the stability and change of youth EA within Chinese society. It further offers several practical implications for promoting higher education enrollment in such a context. The findings demonstrated a strong association between EA during adolescence and subsequent higher education enrollment, and EA trajectories were relatively stable in terms of rank order beginning in early adolescence. This implies that while Chinese students' access to college seems closely tied to their scores on the national college entrance exam, merely improving academic abilities and skills is insufficient for promising enrollment. Teachers and caregivers may wish to invest additional efforts into school and parenting practices aimed at nurturing and sustaining high EA among early adolescents. These efforts may cascade over time, creating enduring patterns of high EA through late adolescence and beyond.

Furthermore, the predictors of developmental trajectories identified in this study offer insights into potential actions and interventions for supporting adolescent EA. For example, given the significant predictive effects of perceived academic competence, teachers and parents may provide more encouragement to boost young people's beliefs in their academic talents and potential. In addition, recognizing the significance of parental educational involvement and parents' expectations, schools and society may strengthen guidance for parents and encourage them to play a more active role in their children's education at home. This might be particularly effective in the Chinese context because of the close‐knit nature of family dynamics. Lastly, considering that low family SES seems to present as a risk factor for persistently low EA in adolescence, tailored supports and interventions should be designed and provided for young people from such backgrounds.

In summary, this study offers empirical evidence highlighting the importance of fostering and sustaining elevated EA in Chinese youth. Fostering high EA early may empower youth to persist and excel in their educational journey, leading to significant benefits for both individuals and families over the long term.

## Conflicts of Interest

The authors declare no conflicts of interest.

## Supporting information


Data S1.

## Data Availability

The analyses presented here were not preregistered. Data, materials, and analytic code are available from the corresponding author upon reasonable request.
