# Supplementary material for: Heterogeneity in the Developmental Trajectories of Chinese Youth Educational Aspirations: Identifying Predictors and Outcomes
Source: Child Dev. 2025 Apr 1;96(3):1220–35. doi: 10.1111/cdev.14234 (PMC12023834; doi:10.1111/cdev.14234)

**Supplementary Materials**

**Table S1**

*Ages and School Stages of The Sample at Each Wave of Data Collection*

| Year | 2010 (T1, N = 2228) | 2012 (T2, N = 1857) | 2014 (N = 1716) |
| --- | --- | --- | --- |
|  |  |  |  |
| Age (*M[SD])* | 11.48[1.12] | 13.48[1.25] | 15.43[1.17] |
| School Stage (%) | Elementary School: 86.18% Middle School: 13.11% Not in school: 0.63% | Elementary School: 30.27% Middle School: 54.38% High School: 3.53% Not in school: 3.48% | Elementary School: 3.32% Middle School: 47.96% High School: 26.86% College (Associate): 0.52% College (Bachelor’s): 0.12% Not in school: 13.17% |
|  |  |  |  |
| Year | 2016 (N=1579) | 2018 (N = 1145) | 2020 (N = 1033) |
|  |  |  |  |
| Age (*M[SD])* | 17.66[1.23] | 19.43[1.12] | 21.42[1.12] |
| School Stage (%) | Elementary School: 0.25% Middle School: 13.24%  High School: 46.23%  College (Associate): 4.62%  College (Bachelor’s): 4.75% Not in school: 30.84% | Elementary School: 0.08% Middle School: 0.96% High School: 28.56% College (Associate): 16.59% College (Bachelor’s): 18.34% Not in school: 35.46% | High School: 4.74% College (Associate): 15.68% College (Bachelor’s): 24.30% Graduate School: 0.68% Not in school: 54.60% |

**Table S2**

*Attrition analysis*

|  | T2 (2012) | | T3 (2014) | | T4 (2016) | |
| --- | --- | --- | --- | --- | --- | --- |
|  | *χ2 (df)* | *t (df)* | *χ2 (df)* | *t (df)* | *χ2 (df)* | *t (df)* |
| Gender (F = 0, M = 1) | 4.58(1)* |  | 1.98(1) |  | 0.17(1) |  |
| Ethnic (Han = 0, Minority = 1) | 2.47(1) |  | 10.82(1)** |  | 3.02(1) |  |
| Residence (Rural = 0, Urban = 1) | 1.70(1) |  | 1.63(1) |  | 5.31(1)* |  |
| Academic Performance |  | 2.16(2210)* |  | 3.85(2210)*** |  | 2.09(2210)* |
| Perceived Academic Competence |  | 0.13(2191) |  | 2.16(2191)* |  | 0.73(2191) |
| Parental Involvement |  | 1.11(1791) |  | 3.09(1791)** |  | 1.58(1791) |
| Parental Academic Expectation |  | 0.90(1776) |  | 3.77(1776)*** |  | 0.41(1776) |
| Parents’ Educational Level |  | -0.94(2165) |  | 2.21(2165)* |  | 0.37(2165) |
| Family Income |  | -1.82(2118) |  | -2.34(2118)* |  | -1.66(2118) |

**p* ≤ .05, ***p* ≤ .01, ****p* ≤ .001. *Note:* Youth with missing EA data (Missing = 1) at T2-T4 were compared to those with complete data (Missing = 0).

**Table S3**

*Descriptive Statistics and Correlation Analyses of The Study*

| Variables | 1 | 2 | 3 | 4 | 5 | 6 | 7 | 8 | 9 | 10 | 11 | 12 | 13 | 14 |
| --- | --- | --- | --- | --- | --- | --- | --- | --- | --- | --- | --- | --- | --- | --- |
| 1 EA 2010 |  |  |  |  |  |  |  |  |  |  |  |  |  |  |
| 2 EA 2012 | 0.36*** |  |  |  |  |  |  |  |  |  |  |  |  |  |
| 3 EA 2014 | 0.34*** | 0.45*** |  |  |  |  |  |  |  |  |  |  |  |  |
| 4 EA 2016 | 0.30*** | 0.39*** | 0.51*** |  |  |  |  |  |  |  |  |  |  |  |
| 5 Gender (F = 0, M = 1) | -0.02 | -0.07** | -0.13*** | -0.18*** |  |  |  |  |  |  |  |  |  |  |
| 6 Ethnicity | -0.11*** | -0.08** | -0.08** | -0.13*** | 0.00 |  |  |  |  |  |  |  |  |  |
| (Han = 0, Minority = 1) |  |  |  |  |  |  |  |  |  |  |  |  |  |  |
| 7 Residence | 0.17*** | 0.17*** | 0.18*** | 0.19*** | 0.02 | -0.11*** |  |  |  |  |  |  |  |  |
| (Rural = 0, Urban = 1) |  |  |  |  |  |  |  |  |  |  |  |  |  |  |
| 8 Aca Perf | 0.27*** | 0.26*** | 0.28*** | 0.32*** | -0.06** | -0.21*** | 0.24*** |  |  |  |  |  |  |  |
| 9 Perceived Aca Comp | 0.23*** | 0.21*** | 0.22*** | 0.22*** | -0.12*** | -0.05* | 0.04 | 0.16*** |  |  |  |  |  |  |
| 10 Par Edu Inv | 0.21*** | 0.22*** | 0.14*** | 0.13*** | 0.04 | -.0.11*** | 0.27*** | 0.22*** | 0.12*** |  |  |  |  |  |
| 11 Par Aca Exp | 0.22*** | 0.20*** | 0.21*** | 0.27*** | -0.07** | -0.16*** | 0.12*** | 0.23*** | 0.19*** | 0.15*** |  |  |  |  |
| 12 Par Edu Level | 0.29*** | 0.28*** | 0.30*** | 0.32*** | -0.03 | -0.17*** | 0.46*** | 0.39*** | 0.13*** | 0.34*** | 0.17*** |  |  |  |
| 13 F Income (¥) | 0.13*** | 0.13*** | 0.17*** | 0.15*** | 0.04 | -0.08*** | 0.30*** | 0.21*** | 0.05** | 0.16*** | 0.08*** | 0.42*** |  |  |
| 14 High Edu Enroll | 0.24*** | 0.32*** | 0.41*** | 0.48*** | -0.16*** | -0.13*** | 0.20*** | 0.39*** | 0.15*** | 0.18*** | 0.20*** | 0.32*** | 0.18*** |  |
| *Mean* | 5.38 | 5.17 | 5.2 | 5.2 | — | — | — | 0 | 3.3 | 3.33 | 90.78 | 2.44 | 6590.05 | — |
| *SD* | 1.5 | 1.33 | 1.26 | 1.25 | — | — | — | 0.87 | 0.77 | 0.9 | 9.29 | 1.05 | 8740.81 | — |

**p* ≤ .05, ***p* ≤ .01, ****p* ≤ .001

*Notes*: 1-4 EA = Educational Aspirations; 8 Aca Perf = Academic Performance; 9 Perceived Aca Comp = Perceived Academic Competence; 10 Par Edu Inv = Parental Education Involvement; 11 Par Aca Exp = Parental academic expectations; 12 Par Edu Level = Parents’ Education Level; 13 F Income = Family Income; 14 High Edu Enroll = Higher Education Enrollment

**Table S4**

*Coefficients for Testing The Interaction Effect of Gender * Urban-Rural Residence on Class Membership*

| Variable | Class 1 vs Class 2 | | | Class 1 vs Class 3 | | | Class 1 vs Class 4 | | | Class 1 vs Class 5 | | | Class 2 vs Class 3 | | |
| --- | --- | --- | --- | --- | --- | --- | --- | --- | --- | --- | --- | --- | --- | --- | --- |
|  | High-decrease vs | | | High-decrease vs | | | High-decrease vs | | | High-decrease vs | | | Moderate-decrease vs | | |
|  | Moderate-decrease | | | Stable | | | Moderate-increase | | | Low-increase | | | Stable | | |
|  | Estimates | SE | p-value | Estimates | SE | p-value | Estimates | SE | p-value | Estimates | SE | p-value | Estimates | SE | p-value |
| Gender (F = 0, M = 1) | -0.38 | 0.17 | 0.03 | -0.20 | 0.24 | 0.40 | -0.01 | 0.18 | 0.94 | 0.00 | 0.21 | 1.00 | 0.18 | 0.21 | 0.40 |
| Residence (Rural = 0, Urban = 1) | -0.30 | 0.18 | 0.11 | -0.77 | 0.29 | 0.01 | -0.80 | 0.21 | 0.00 | -1.40 | 0.29 | 0.00 | -0.48 | 0.26 | 0.06 |
| Gender * Residence | 0.22 | 0.25 | 0.38 | 0.27 | 0.39 | 0.49 | 0.06 | 0.29 | 0.83 | 0.38 | 0.38 | 0.31 | 0.05 | 0.36 | 0.90 |
|  |  |  |  |  |  |  |  |  |  |  |  |  |  |  |  |
| Variable | Class 2 vs Class 4 | | | Class 2 vs Class 5 | | | Class 3 vs Class 4 | | | Class 3 vs Class 5 | | | Class 4 vs Class 5 | | |
|  | Moderate-decrease vs | | | Moderate-decrease vs | | | Stable vs | | | Stable vs | | | Moderate-increase vs | | |
|  | Moderate-increase | | | High-increase | | | Moderate-increase | | | High-increase | | | High-increase | | |
|  | Estimates | SE | p-value | Estimates | SE | p-value | Estimates | SE | p-value | Estimates | SE | p-value | Estimates | SE | p-value |
| Gender (F = 0, M = 1) | 0.36 | 0.14 | 0.01 | 0.38 | 0.17 | 0.03 | 0.19 | 0.22 | 0.39 | 0.20 | 0.24 | 0.40 | 0.01 | 0.18 | 0.95 |
| Residence (Rural = 0, Urban = 1) | -0.50 | 0.17 | 0.00 | -1.11 | 0.26 | 0.00 | -0.02 | 0.28 | 0.94 | -0.63 | 0.34 | 0.07 | -0.61 | 0.29 | 0.03 |
| Gender * Residence | -0.16 | 0.24 | 0.51 | 0.16 | 0.34 | 0.64 | -0.21 | 0.39 | 0.59 | 0.11 | 0.45 | 0.80 | 0.32 | 0.37 | 0.39 |

*Note*: No significant gender * residence interaction effect was found

## **Sensitivity Analyses**

Given the complexity of the modeling procedures in the current study, we conducted a series of sensitivity analyses to ensure the robustness of our findings.

First, in our primary analysis, we fixed the intercept variance of the 5-trajectory class-invariant model to zero to address the Heywood case of negative variance that appeared in the initial specification. This restriction was applied across all potential solutions to utilize the Likelihood ratio tests (LRT) for model comparisons. Here, we re-ran the model selection procedure without imposing the zero-intercept variance restriction to assess whether it would significantly alter the results (see Tables S5 and S6, and Figure S1).

Second, we explored alternative approaches for handling missing data in EA and predictor variables (except gender, ethnicity, and rural-nonrural residence) by employing multiple imputations (MI) with 50 datasets based on a Bayesian estimation of an unrestricted model in Mplus 8 (Asparouhov & Muthén, 2022). Given that the LRT tests did not apply to datasets generated through multiple imputations in Mplus, we specified and tested only the 5-trajectory class-invariant model, and then proceeded with the trajectory prediction analysis (see Tables S7 and S8, and Figure S2). We opted not to impute higher education enrollment status, as we deemed it logically inappropriate to impute an outcome variable using other variables and then predict the outcome.

Third, we repeated the trajectory identification procedure with a subsample that included only adolescents who were in elementary school when the study began in 2010 (comprising 86.18% of the entire sample). This was done to verify whether the initial school stage would substantially impact the youth's EA development trajectory (see Tables S9 and S10, and Figure S3).

The results from these three sets of analyses suggest that we largely replicated the original findings presented in the main manuscript. This suggests that the restrictions on zero-intercept variance, the methods for handling missing data, and the initial school stage of the sample did not substantially influence the model structure, fit, and the substantive implications of the results.

Finally, in our primary analysis, we utilized several categorical variables to predict trajectory class memberships. This approach inherently assumed that the number and characteristics of trajectory classes were consistent across all levels of the categorical variable. To assess the validity of this assumption, we conducted a separate trajectory identification process for boys and girls, as the sample sizes for both genders were sufficiently large to establish classes with meaningful substantive interpretations. The results indicated that, for boys, a 5-trajectory class-invariant model, identical to the optimal solution in the overall sample (including estimated coefficients and class percentages), was also identified as the best representation of the EA development sub-trajectories (see Tables S11 and S12, and Figure S4). For girls, however, a 4-trajectory class-invariant model emerged as the optimal solution, with Classes 3 and 4 in the whole-sample 5-trajectory model merging into a single class (see Tables S13 and S14, and Figure S5). A higher percentage of individuals belonged to classes that started with aspirations to pursue higher education, which fluctuated over the years but remained above an associate degree (i.e., Class 1 and 2 in the 5-trajectory model). Despite these, the overall pattern of a considerable initial dispersion followed by gradual convergence and divergence remained evident across the trajectories. Therefore, we do not consider the two gender groups to be qualitatively different in their EA development.

It is worth noting, however, that though we have re-conducted the trajectory identification process separately for boys and girls, similar procedures are not feasible for ethnicity and urban-rural residence due to insufficient sample sizes in each subgroup. Future research could address this limitation by oversampling youth from specific demographic groups.

**Sensitivity Analysis 1: Removing the Zero-Intercept-Variance Restriction**

**Table S5**

*Fit Indices of 1-trajectory to 7-trajectory Model Without the Zero-Intercept-Variance Restriction*

| Group | Log-likelihood | AIC | BIC | ASBIC | VLMR-LRT | LMR-LRT | BLRT | Entropy |
| --- | --- | --- | --- | --- | --- | --- | --- | --- |
| 1 | -10999.69 | 22025.39 | 22099.60 | 22058.30 | N/A | N/A | N/A | N/A |
| 2 | -10872.57 | 21779.13 | 21876.18 | 21822.17 | <.001 | <.001 | <.001 | 0.62 |
| 3 | -10629.50 | 21301.01 | 21420.89 | 21354.17 | <.001 | <.001 | <.001 | 0.92 |
| 4 | -10459.09 | 20968.18 | 21110.90 | 21031.47 | <.001 | <.001 | <.001 | 0.91 |
| **5** | **-10177.61** | **20413.21** | **20578.77** | **20486.63** | **<.005** | **<.005** | **<.001** | **0.98** |
| 6 | -9203.39 | 18472.79 | 18661.18 | 18556.33 | >.05 | >.05 | <.001 | 0.98 |
| 7 | -6627.09 | 13328.19 | 13539.41 | 13421.86 | >.05 | >.05 | <.001 | 0.98 |

**Table S6**

| Parameter | Class 1 | Class 2 | Class 3 | Class 4 | Class 5 |
| --- | --- | --- | --- | --- | --- |
| Mean |  |  |  |  |  |
| Intercept | 7.62*** | 6.00*** | 5.00*** | 4.01*** | 2.85*** |
| Linear slope | -1.92*** | -0.68*** | 0.15 | 0.80*** | 1.39*** |
| Quadratic slope | 0.42*** | 0.17*** | -0.04 | -0.17*** | -0.32*** |
| Variances |  |  |  |  |  |
| Intercept | -0.04 | -0.04 | -0.04 | -0.04 | -0.04 |
| Linear slope | 0.65*** | 0.65*** | 0.65*** | 0.65*** | 0.65*** |
| Quadratic slope | 0.04* | 0.04* | 0.04* | 0.04* | 0.04* |
| Covariances |  |  |  |  |  |
| Intercept, linear slope | 0.05 | 0.05 | 0.05 | 0.05 | 0.05 |
| Intercept, quadratic slop | -0.01 | -0.01 | -0.01 | -0.01 | -0.01 |
| Linear slope, quadratic slope | -0.15*** | -0.15*** | -0.15*** | -0.15*** | -0.15*** |

*Parameter Estimates for the Selected Five-Class Growth Mixture Model Without the Zero-Intercept-Variance Restriction*

** p < .05. **p < .01. ***p < .001.*

*Note:* Since the negative variance is not significant and its absolute value is small, removing the restricting did not substantially change the parameter estimates.

**Figure S1**

*Trajectories of The Five Latent Classes Without the Zero-Intercept-Variance Restriction*


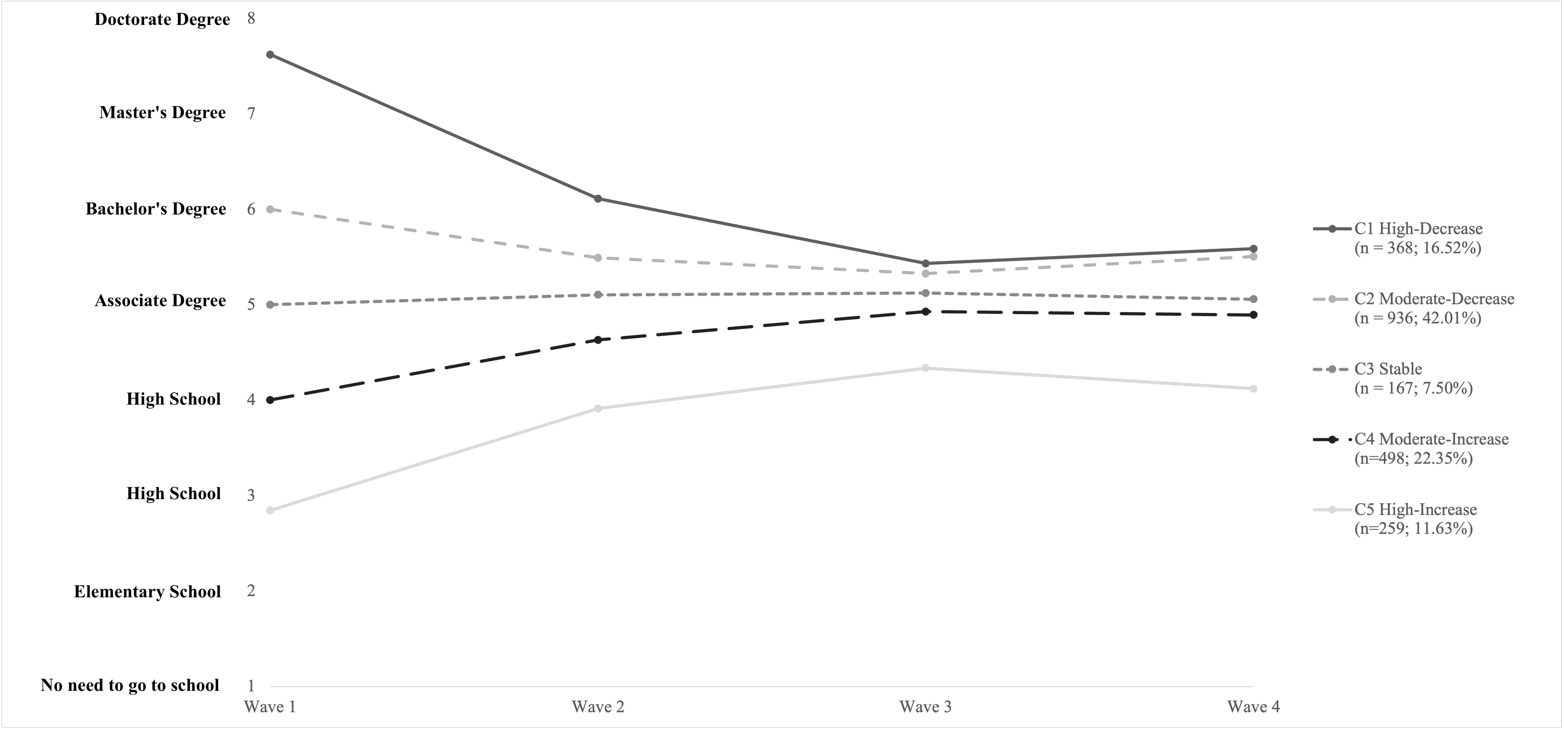


*Note:* This figure is nearly the same as the one from our primary analysis.

**Sensitivity Analysis 2: Employing Multiple Imputations (MI) to Handle Missing Data in EA and Predictors**

**Table S7**

| Parameter | Class 1 | Class 2 | Class 3 | Class 4 | Class 5 |
| --- | --- | --- | --- | --- | --- |
| Mean |  |  |  |  |  |
| Intercept | 7.62*** | 6.00*** | 5.00*** | 4.00*** | 2.84*** |
| Linear slope | -1.86*** | -0.65*** | 0.07 | 0.77*** | 1.33*** |
| Quadratic slope | 0.40*** | 0.16*** | -0.02 | -0.16*** | -0.29*** |
| Variances |  |  |  |  |  |
| Intercept | 0.00 | 0.00 | 0.00 | 0.00 | 0.00 |
| Linear slope | 0.68*** | 0.68*** | 0.68*** | 0.68*** | 0.68*** |
| Quadratic slope | 0.04* | 0.04* | 0.04* | 0.04* | 0.04* |
| Covariances |  |  |  |  |  |
| Linear slope, quadratic slope | -0.16*** | -0.16*** | -0.16*** | -0.16*** | -0.16*** |

*Parameter Estimates for the Selected Five-Class Growth Mixture Model With Datasets from Multiple Imputations*

** p < .05. **p < .01. ***p < .001.*

**Figure S2**

*Trajectories of The Five Latent Classes With Datasets from Multiple Imputations*

**
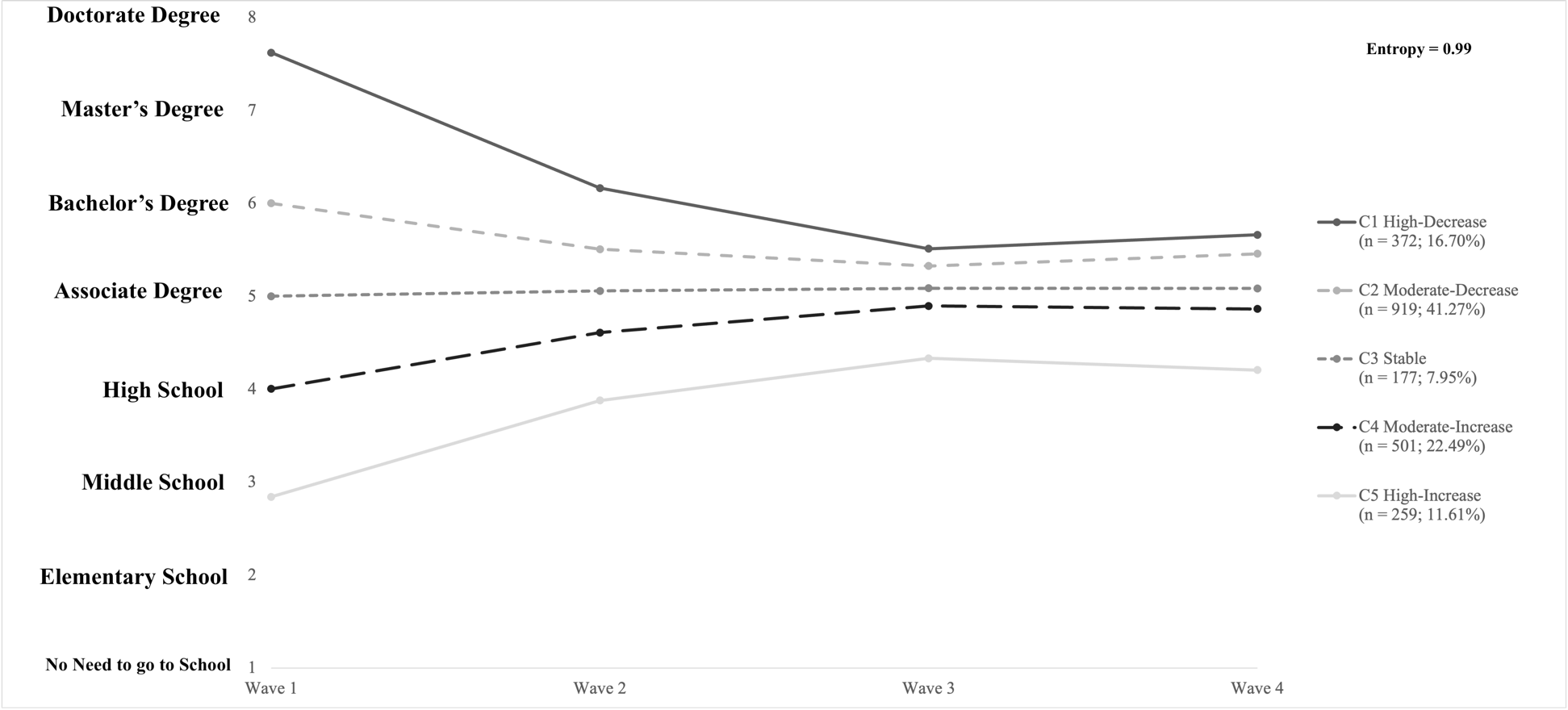
**

**Table S8**

*The Associations Between Individual and Family Contextual Factors and Class Membership With Datasets from Multiple Imputations*

| Variable | Class 1 vs Class 2 | | | Class 1 vs Class 3 | | | Class 1 vs Class 4 | | | Class 1 vs Class 5 | | | Class 2 vs Class 3 | | |
| --- | --- | --- | --- | --- | --- | --- | --- | --- | --- | --- | --- | --- | --- | --- | --- |
|  | Estimates | SE | OR | Estimates | SE | OR | Estimates | SE | OR | Estimates | SE | OR | Estimates | SE | OR |
| Gender (F = 0, M = 1) | **-0.32*** | **0.13** | **0.73** | -0.21 | 0.19 | 0.81 | -0.14 | 0.15 | 0.87 | -0.19 | 0.18 | 0.83 | 0.11 | 0.17 | 1.12 |
| Ethnic (Han = 0, Minority = 1) | -0.07 | 0.23 | 0.94 | 0.47 | 0.30 | 1.60 | -0.13 | 0.25 | 0.88 | 0.04 | 0.28 | 1.04 | 0.54 | 0.25 | 1.71 |
| Residence (Rural = 0, Urban = 1) | 0.18 | 0.15 | 1.19 | -0.15 | 0.23 | 0.86 | -0.11 | 0.17 | 0.89 | 0.11 | 0.22 | 1.12 | -0.33 | 0.20 | 0.72 |
| Academic Performance | -0.17 | 0.09 | 0.85 | 0.03 | 0.14 | 1.03 | **-0.28**** | **0.10** | **0.75** | **-0.72***** | **0.12** | **0.49** | 0.20 | 0.13 | 1.22 |
| Perceived Academic Competence | **-0.25**** | **0.07** | **0.78** | **-0.38***** | **0.10** | **0.68** | **-0.41***** | **0.08** | **0.66** | **-0.73***** | **0.10** | **0.48** | -0.13 | 0.08 | 0.88 |
| Parental Involvement | **-0.23**** | **0.08** | **0.80** | **-0.31*** | **0.11** | **0.74** | **-0.28**** | **0.09** | **0.76** | **-0.47***** | **0.11** | **0.63** | -0.08 | 0.10 | 0.92 |
| Parental Academic Expectation | -0.16 | 0.08 | 0.85 | -0.22 | 0.12 | 0.81 | **-0.34***** | **0.09** | **0.71** | **-0.39***** | **0.10** | **0.68** | -0.05 | 0.10 | 0.95 |
| Parents’ Educational Level | **-0.20*** | **0.08** | **0.82** | **-0.36**** | **0.12** | **0.69** | **-0.44***** | **0.10** | **0.65** | **-0.78***** | **0.14** | **0.46** | -0.17 | 0.11 | 0.85 |
| Family Income | 0.04 | 0.06 | 1.04 | 0.09 | 0.09 | 1.09 | 0.04 | 0.10 | 1.04 | -0.31 | 0.18 | 0.74 | 0.05 | 0.07 | 1.05 |
|  |  |  | |  |  |  |  |  |  |  |  |  |  |  |  |
| Variable | Class 2 vs Class 4 | | | Class 2 vs Class 5 | | | Class 3 vs Class 4 | | | Class 3 vs Class 5 | | | Class 4 vs Class 5 | | |
|  | Estimates | SE | OR | Estimates | SE | OR | Estimates | SE | OR | Estimates | SE | OR | Estimates | SE | OR |
| Gender (F = 0, M = 1) | 0.18 | 0.12 | 1.2 | 0.13 | 0.16 | 1.14 | 0.07 | 0.18 | 1.07 | 0.02 | 0.21 | 1.02 | -0.05 | 0.17 | 0.96 |
| Ethnic (Han = 0, Minority = 1) | -0.06 | 0.19 | 0.94 | 0.11 | 0.22 | 1.11 | **-0.60*** | **0.26** | **0.55** | -0.43 | 0.29 | 0.65 | 0.17 | 0.23 | 1.19 |
| Residence (Rural = 0, Urban = 1) | **-0.29*** | **0.14** | **0.75** | -0.07 | 0.19 | 0.94 | 0.04 | 0.22 | 1.04 | 0.26 | 0.26 | 1.30 | 0.22 | 0.21 | 1.25 |
| Academic Performance | -0.12 | 0.07 | 0.89 | **-0.56***** | **0.10** | **0.57** | **-0.31*** | **0.13** | **0.73** | **-0.75***** | **0.14** | **0.47** | **-0.44***** | **0.10** | **0.64** |
| Perceived Academic Competence | **-0.16*** | **0.06** | **0.85** | **-0.48***** | **0.08** | **0.62** | -0.03 | 0.09 | 0.97 | **-0.35***** | **0.10** | **0.70** | **-0.32***** | **0.08** | **0.73** |
| Parental Involvement | -0.05 | 0.07 | 0.95 | **-0.24*** | **0.09** | **0.79** | 0.03 | 0.11 | 1.03 | -0.16 | 0.12 | 0.85 | **-0.19*** | **0.09** | **0.83** |
| Parental Academic Expectation | **-0.18*** | **0.06** | **0.84** | **-0.23**** | **0.08** | **0.80** | -0.12 | 0.1 | 0.89 | -0.18 | 0.11 | 0.84 | -0.05 | 0.08 | 0.95 |
| Parents’ Educational Level | **-0.24**** | **0.08** | **0.78** | **-0.58***** | **0.12** | **0.56** | -0.08 | 0.12 | 0.93 | **-0.41*** | **0.15** | **0.66** | **-0.34*** | **0.13** | **0.71** |
| Family Income | 0.00 | 0.09 | 1.00 | -0.35 | 0.18 | 0.71 | -0.05 | 0.11 | 0.96 | **-0.39*** | **0.19** | **0.68** | -0.35 | 0.19 | 0.71 |

**p* ≤ .05, ***p* ≤ .01, ****p* ≤ .001

*Note:* Reference classes are listed on the left side

**Sensitivity Analysis 3: Repeating the Class Identification Process for A Subsample of Elementary School Participants in 2010 (N = 1920)**

**Table S9**

*Fit Indices of 1-trajectory to 6-trajectory Model for Elementary Stage Participants*

| Group | Log-likelihood | AIC | BIC | ASBIC | VLMR-LRT | LMR-LRT | BLRT | Entropy |
| --- | --- | --- | --- | --- | --- | --- | --- | --- |
| 1 | -9694.209 | 19408.418 | 19464.019 | 19432.249 | N/A | N/A | N/A | N/A |
| 2 | -9491.942 | 19011.883 | 19089.725 | 19045.246 | <.001 | <.001 | <.001 | 0.79 |
| 3 | -9257.480 | 18550.961 | 18651.042 | 18593.856 | <.001 | <.001 | <.001 | 0.92 |
| 4 | -9065.919 | 18175.837 | 18298.159 | 18228.265 | <.001 | <.001 | <.001 | 0.91 |
| **5** | **-8850.376** | **17752.752** | **17897.315** | **17814.712** | **<.001** | **<.001** | **<.001** | **0.97** |
| 6 | -8077.155 | 16214.309 | 16381.111 | 16285.801 | >.05 | >.05 | **<.001** | 0.98 |
| 6 | -5906.154 | 11880.308 | 12069.351 | 11961.332 | >.05 | >.05 | <.001 | 0.98 |

**Table S10**

| Parameter | Class 1 | Class 2 | Class 3 | Class 4 | Class 5 |
| --- | --- | --- | --- | --- | --- |
| Mean |  |  |  |  |  |
| Intercept | 7.65*** | 6.00*** | 5.00*** | 4.00*** | 2.84*** |
| Linear slope | -1.96*** | -0.71*** | 0.16 | 0.82*** | 1.41*** |
| Quadratic slope | 0.41*** | 0.18*** | -0.05 | -0.16*** | -0.33*** |
| Variances |  |  |  |  |  |
| Intercept | 0.00 | 0.00 | 0.00 | 0.00 | 0.00 |
| Linear slope | 0.72*** | 0.72*** | 0.72*** | 0.72*** | 0.72*** |
| Quadratic slope | 0.04* | 0.04* | 0.04* | 0.04* | 0.04* |
| Covariances |  |  |  |  |  |
| Linear slope, quadratic slope | -0.17*** | -0.17*** | -0.17*** | -0.17*** | -0.17*** |

*Parameter Estimates for the Selected Five-Class Growth Mixture Model for* *Elementary Stage Participants*

** p < .05. **p < .01. ***p < .00*

**Figure S3**

*Trajectories of The Five Latent Classes for Elementary Stage Participants*

*
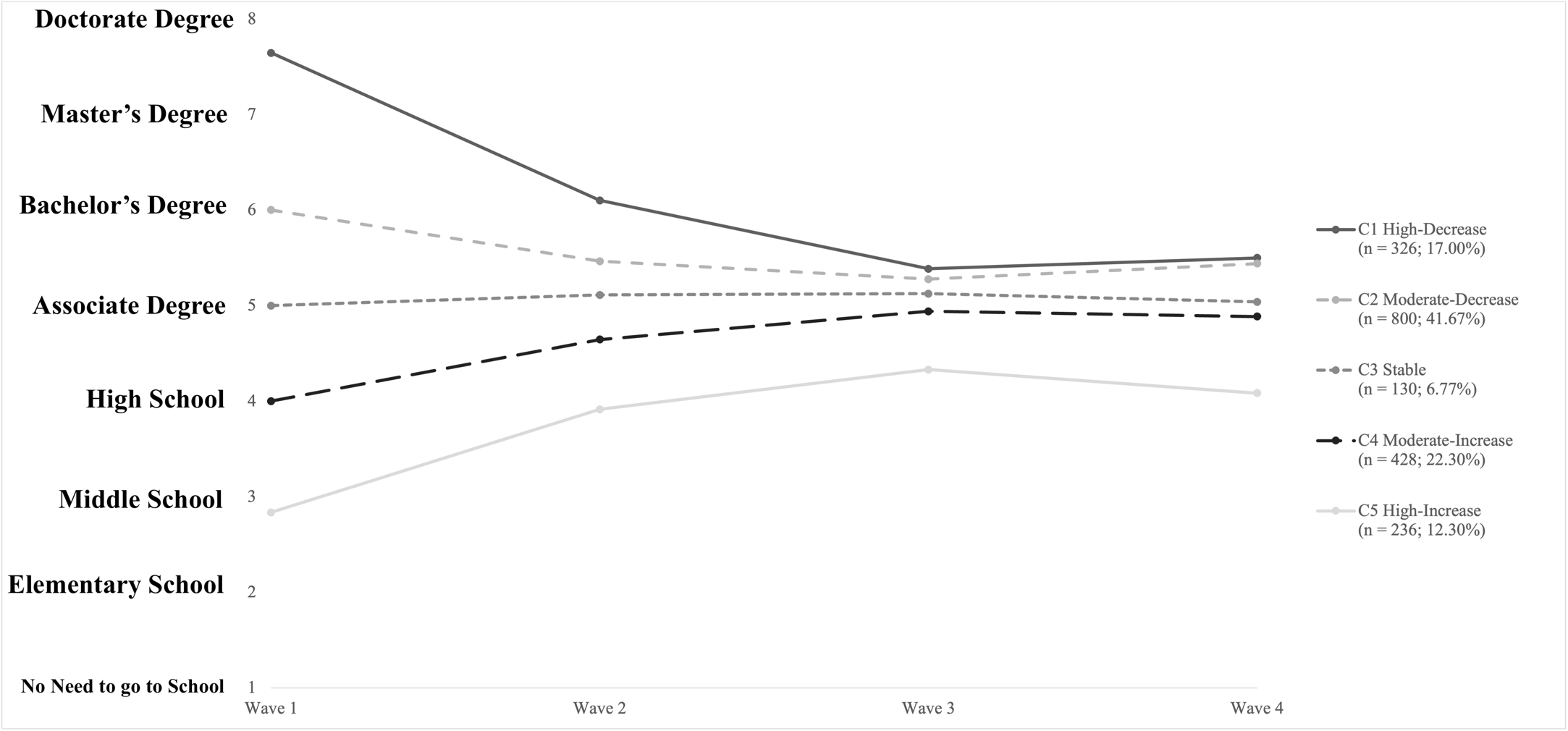
*

**Sensitivity Analysis 4: Repeating the Class Identification Process Separately for Male and Female**

**Sensitivity Analysis 4.1 Male (N = 1145)**

**Table S11**

*Fit Indices of 1-trajectory to 7-trajectory Model for Male Youth*

| Group | Log-likelihood | AIC | BIC | ASBIC | VLMR-LRT | LMR-LRT | BLRT | Entropy |
| --- | --- | --- | --- | --- | --- | --- | --- | --- |
| 1 | -5813.72 | 11647.43 | 11697.87 | 11666.10 | N/A | N/A | N/A | N/A |
| 2 | -5693.83 | 11415.66 | 11486.27 | 11441.80 | <.001 | <.001 | <.001 | 0.64 |
| 3 | -5548.28 | 11132.56 | 11223.34 | 11166.16 | <.001 | <.001 | <.001 | 0.93 |
| 4 | -5440.02 | 10924.04 | 11034.99 | 10965.11 | <.001 | <.001 | <.001 | 0.92 |
| **5** | **-5275.36** | **10602.72** | **10733.84** | **10651.25** | **<.01** | **<.01** | **<.001** | **0.98** |
| 6 | -4713.74 | 9487.49 | 9638.78 | 9543.49 | >.05 | >.05 | <.001 | 0.99 |
| 7 | -3093.39 | 6254.79 | 6426.25 | 6318.26 | >.05 | >.05 | <.001 | 0.99 |

**Table S12**

*Parameter Estimates for the Selected Five-Class Growth Mixture Model for Male Youth*

| Parameter | Class 1 | Class 2 | Class 3 | Class 4 | Class 5 |
| --- | --- | --- | --- | --- | --- |
| Mean |  |  |  |  |  |
| Intercept | 7.66*** | 6.00*** | 5.00*** | 4.00*** | 2.87*** |
| Linear slope | -2.00*** | -0.80*** | 0.13 | 0.75*** | 1.15*** |
| Quadratic slope | 0.41*** | 0.19*** | -0.06 | -0.17*** | -0.26*** |
| Variances |  |  |  |  |  |
| Intercept | 0.00 | 0.00 | 0.00 | 0.00 | 0.00 |
| Linear slope | 0.74*** | 0.74*** | 0.74*** | 0.74*** | 0.74*** |
| Quadratic slope | 0.03 | 0.03 | 0.03 | 0.03 | 0.03 |
| Covariances |  |  |  |  |  |
| Linear slope, quadratic slope | -0.17** | -0.17** | -0.17** | -0.17** | -0.17** |

** p < .05. **p < .01. ***p < .001.*

**Figure S4**

*Trajectories of The Five Latent Classes for Male Youth*

*
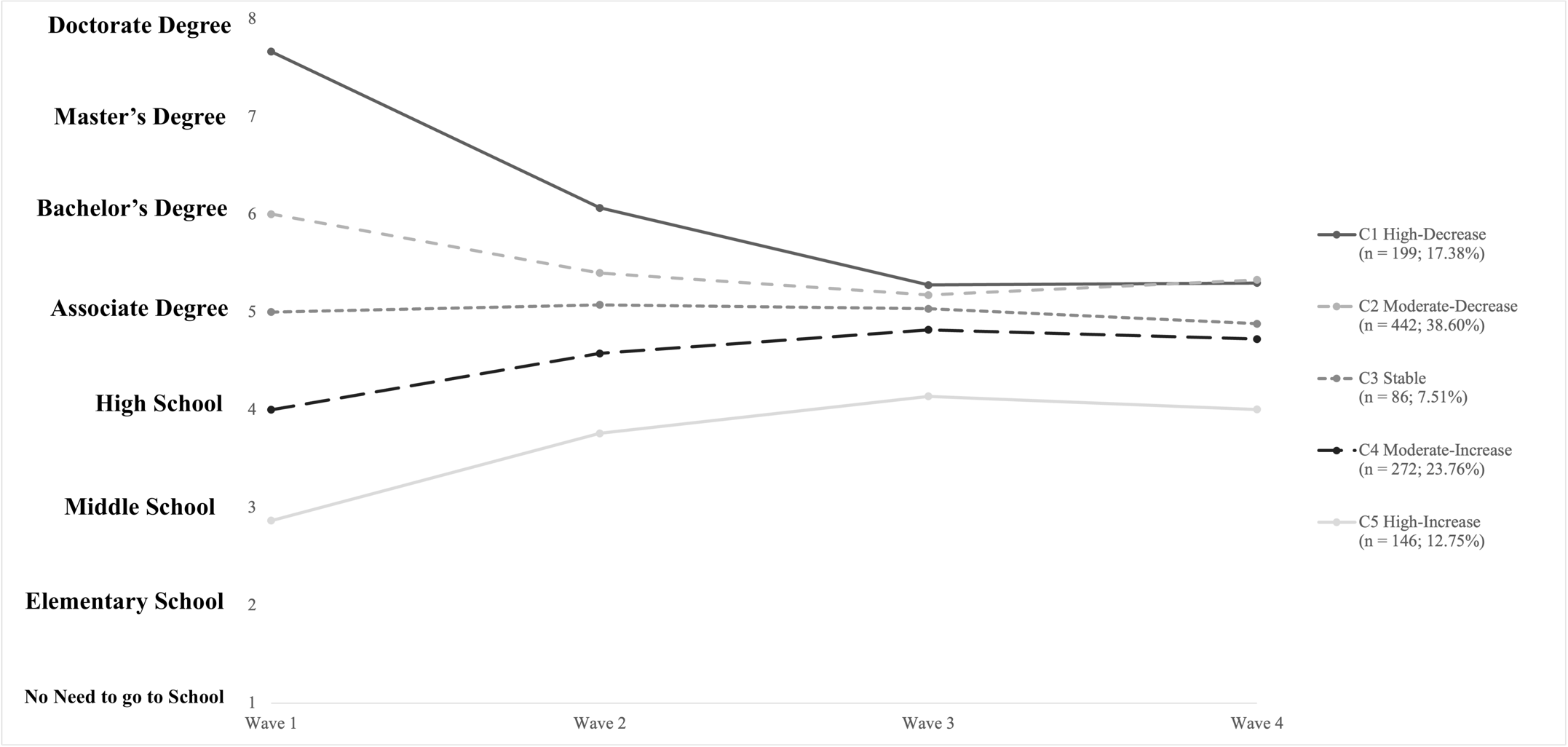
*

**Sensitivity Analysis 4.2 Female (N = 1083)**

**Table S13**

*Fit Indices of 1-trajectory to 6-trajectory Model for Female Youth*

| Group | Log-likelihood | AIC | BIC | ASBIC | VLMR-LRT | LMR-LRT | BLRT | Entropy |
| --- | --- | --- | --- | --- | --- | --- | --- | --- |
| 1 | -5309.51 | 10639.03 | 10688.90 | 10657.14 | N/A | N/A | N/A | N/A |
| 2 | -5177.22 | 10382.45 | 10452.27 | 10407.81 | <.001 | <.001 | <.001 | 0.70 |
| 3 | -5065.65 | 10167.31 | 10257.08 | 10199.91 | <.001 | <.001 | <.001 | 0.91 |
| **4** | **-4966.95** | **9977.89** | **10087.62** | **10017.74** | **<.001** | **<.001** | **<.001** | **0.91** |
| 5 | -4840.62 | 9733.25 | 9862.92 | 9780.34 | >.05 | >.05 | <.001 | 0.98 |
| 6 | -4420.13 | 8900.26 | 9049.89 | 8954.60 | >.05 | >.05 | <.001 | 0.98 |

**Table S14**

| Parameter | Class 1 | Class 2 | Class 3 | Class 4 |
| --- | --- | --- | --- | --- |
| Mean |  |  |  |  |
| Intercept | 7.63*** | 5.93*** | 4.11*** | 2.82*** |
| Linear slope | -1.89*** | -0.50*** | 0.74*** | 1.63*** |
| Quadratic slope | 0.44*** | 0.13*** | -0.14*** | -0.39*** |
| Variances |  |  |  |  |
| Intercept | 0.00 | 0.00 | 0.00 | 0.00 |
| Linear slope | 0.64*** | 0.64*** | 0.64*** | 0.64*** |
| Quadratic slope | 0.05* | 0.05* | 0.05* | 0.05* |
| Covariances |  |  |  |  |
| Linear slope, quadratic slope | -0.17** | -0.17** | -0.17** | -0.17** |

*Parameter Estimates for the Selected Five-Class Growth Mixture Model for Female Youth*

** p < .05. **p < .01. ***p < .001.*

**Figure S5**

*Trajectories of The Four Latent Classes for Female Youth*


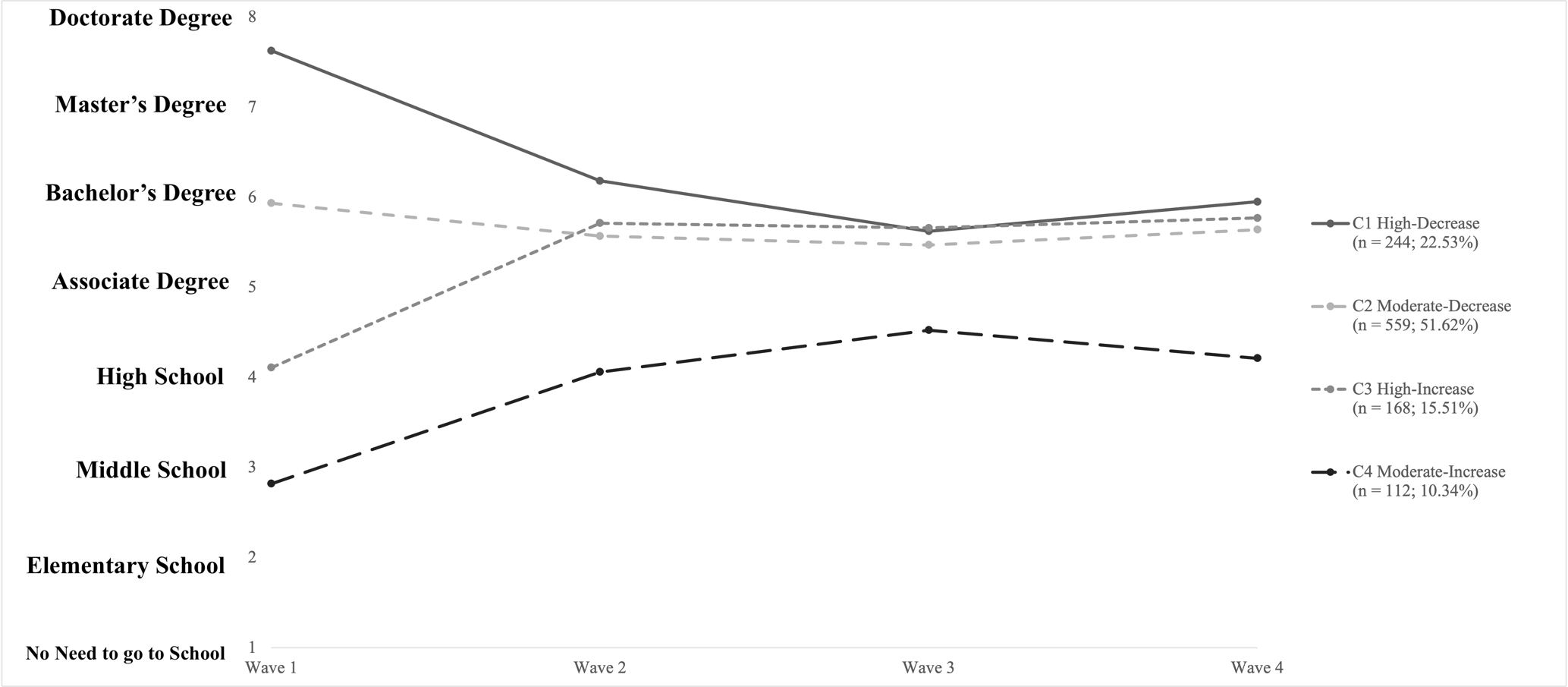

Supplement: Supplementary file 1 — Data S1. [file CDEV-96-1220-s001.docx]
